# Rotary properties of hybrid F_1_-ATPases consisting of subunits from different species

**DOI:** 10.1016/j.isci.2023.106626

**Published:** 2023-04-08

**Authors:** Ryo R. Watanabe, Busra Tas Kiper, Mariel Zarco-Zavala, Mayu Hara, Ryohei Kobayashi, Hiroshi Ueno, José J. García-Trejo, Chun-Biu Li, Hiroyuki Noji

**Affiliations:** 1 Department of Applied Chemistry, Graduate School of Engineering, The University of Tokyo, Tokyo 113-8656, Japan; 2 Department of Mathematics, Stockholm University, 106 91 Stockholm, Sweden; 3 Department of Biology, Chemistry Faculty, National Autonomous University of Mexico, Mexico 04510, Mexico

**Keywords:** Biophysics, Cell biology, Structural biology

## Abstract

F_1_-ATPase (F_1_) is an ATP-driven rotary motor protein ubiquitously found in many species as the catalytic portion of F_o_F_1_-ATP synthase. Despite the highly conserved amino acid sequence of the catalytic core subunits: α and β, F_1_ shows diversity in the maximum catalytic turnover rate *V*_max_ and the number of rotary steps per turn. To study the design principle of F_1_, we prepared eight hybrid F_1_s composed of subunits from two of three genuine F_1_s: thermophilic *Bacillus* PS3 (TF_1_), bovine mitochondria (*b*MF_1_), and *Paracoccus denitrificans* (PdF_1_), differing in the *V*_max_ and the number of rotary steps. The *V*_max_ of the hybrids can be well fitted by a quadratic model highlighting the dominant roles of β and the couplings between α-β. Although there exist no simple rules on which subunit dominantly determines the number of steps, our findings show that the stepping behavior is characterized by the combination of all subunits.

## Highlights

• Preparation of hybrid F_1_-ATPases consisting of subunits from different species
• Maintaining rotary catalysis of F_1_-ATPase with replacement of catalytic core subunit
• Dominant effect of the coupling of catalytic core subunits on the rotation rate of F_1_
• Nontrivial interplays among all subunits for determining the rotation scheme of F_1_

## Graphical abstract

**Figure:**
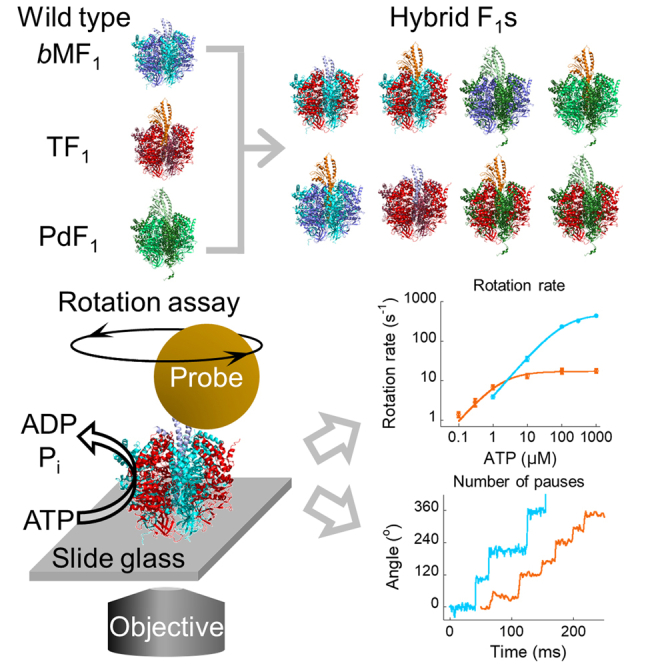

## Introduction

F_o_F_1_-ATP synthase (F_o_F_1_) is the enzyme responsible for the terminal reaction of oxidative phosphorylation, ATP synthesis coupled with proton translocation along proton motive force (*pmf*) across biological membranes.^1^^,^^2^ As indicated in the name, F_o_F_1_ is the complex of two distinctive portions, F_o_ and F_1_, both of which work as rotary molecular motors. F_o_ undergoes rotation of its oligomeric rotor ring driven by proton translocation along *pmf*. F_1_ is an ATP-driven rotary motor whose central shaft rotor rotates against the surrounding catalytic stator ring. F_o_ and F_1_ work together to interconvert *pmf* to the free energy of ATP hydrolysis.

The α_3_β_3_γ subcomplex of F_1_ is the minimum ATP-driven rotary complex in which the rotor shaft, the γ subunit, is rotated against the catalytic α_3_β_3_ stator ring upon ATP hydrolysis.^3^^,^^4^ The α_3_β_3_ stator ring possesses six ATP binding sites at the α-β interfaces, three of which are catalytic while the other three are non-catalytic. Catalytic residues are almost all on the β subunit,^5^^,^^6^^,^^7^ while the α subunit has one arginine residue critical for catalysis, termed the arginine finger,^8^ which stabilizes the transition state of ATP hydrolysis.^9^ The γ subunit consists of a coiled coil of the N- and C-terminal helices that is held in the central cavity of the α_3_β_3_-ring, and a globular domain that protrudes from the α_3_β_3_-ring. In a whole complex of F_o_F_1_, the globular domain of the γ subunit binds to the rotor ring of the F_o_ motor, forming the rotor complex. F_1_ has other minor subunits. In bacterial systems, F_1_ has the δ and ε subunits, while in mitochondrial systems, F_1_ (MF_1_) has a different composition of the minor subunits (Figure S1). In both systems, the minor subunits are structural proteins to build the whole complex of F_o_F_1_ and are not directly involved in the catalysis.^10^ An exception is the bacterial ε subunit that acts as a suppressive regulator in some species by modulating the duration time of catalytically inactive state of F_1_.^11^ Nevertheless, the ε subunit has only minor effect on catalysis and rotary behaviors when F_1_ is active for catalysis.^12^^,^^13^^,^^14^ Therefore, we only focus on the roles of α, β, and γ subunits and simply refer to the α_3_β_3_γ, the α_3_β_3_γδε subcomplexes as well as the whole F_1_ complex as F_1_ hereafter.

Chemo-mechanical pathways of F_1_ have been well characterized by single-molecule rotary assay,^3^^,^^15^^,^^16^^,^^17^^,^^18^^,^^19^^,^^20^ together with biochemical and structural studies, which set the groundwork for theoretical studies.^21^^,^^22^ In a standard rotary assay, F_1_ is immobilized on a glass surface functionalized with nickel-nitrilotriacetic acid (NTA) through histidine-tags of the α_3_β_3_ stator ring. To visualize the rotation of the rotor γ subunit, a rotary marker probe, such as polystyrene beads, magnetic beads, gold colloid, or nanorod, is attached to the protruding domain of the γ subunit and observed under optical microscopes. The principal approach in elucidating the reaction scheme is to resolve the rotations into discrete rotary steps, each intervened by pauses due to rate-determining catalytic states. Under ATP-limiting conditions, F_1_ shows 120° steps intervened by ATP-waiting pause (*binding dwell*). When the ATP hydrolysis step is retarded by mutations of a catalytic residue or by the use of hydrolyzable ATP analog, such as the ATPγS, F_1_ shows 120° steps with catalysis-waiting pauses (*catalytic dwell*). By analyzing both the *binding* and *catalytic dwell* in single F_1_ molecules, one can then determine the angular positions of the *catalytic dwells* relative to the *binding dwells*, to determine the size of substeps triggered by ATP binding and catalysis, respectively.

**Figure 1:**
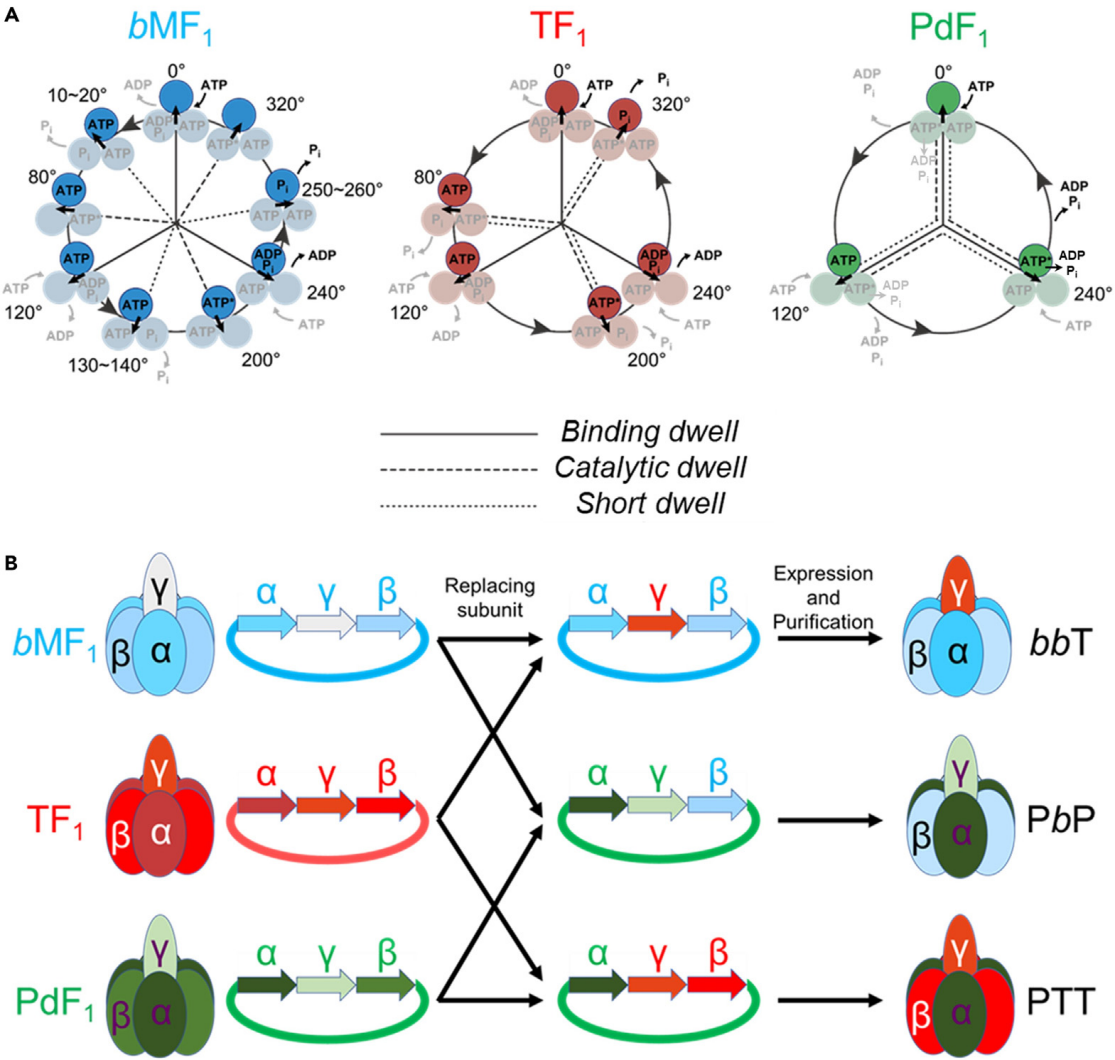
Concept of this study (A) Rotation schemes of genuine F_1_s as previously determined. Each circle and black arrow represent one β subunit and angular position of the γ. 0° is defined as the ATP-binding position at the highlighted β. ATP∗ represents pre- or post-hydrolysis state of bound ATP. (B) Construction of representative hybrid F_1_s. The plasmid was expressed in *Escherichia coli* and the purification of the target protein was performed in the same manner as previously described^26^ for TF_1_-*b*MF_1_ hybrids,^27^ for PdF_1_ hybrids.

F_1_ from thermophilic *Bacillus* PS3 (TF_1_), the best characterized F_1_ in rotary assay, shows 2 substeps of 80° and 40° in a 120° rotation,^5^^,^^16^ initiated after the *binding* and the *catalytic dwell*, respectively. The middle panel in Figure 1A shows the reaction scheme of TF_1_. Each catalytic site hydrolyzes one ATP molecule and couples with one turn of the γ subunit. Although each catalytic site follows exactly the same reaction process, the phase of reaction states differs by 120° among the three catalytic sites. Specifically, each catalytic site cleaves bound ATP into ADP and P_i_ (inorganic phosphate) after a 200° rotation from the angle where the ATP is bound to the catalytic site.^18^ The catalytic site then releases the ADP and the P_i_ at 240°^19^ and 320°,^20^ respectively (Figure 1A middle panel, Table S1). Although the angular position of each elementary reaction remains to be determined, the reaction schemes of the F_1_’s from *Escherichia coli* (EF_1_)^23^ and the *yeast* mitochondria (*y*MF_1_)^24^ are shared with TF_1_. Nonetheless, recent studies revealed the diversity of the reaction scheme of F_1_. In particular, mitochondrial F_1_ from human^25^ or bovine mitochondria (*b*MF_1_)^26^ has an extra pause, termed the “*short dwell*”, in addition to the *binding* and *catalytic dwells* (Figure 1A left panel). On the other hand, F_1_ from *Paracoccus denitrificans* (PdF_1_) has its *binding* and *catalytic dwells* at the same angles, showing that PdF_1_ has only 3 steps regardless of the ATP concentration, [ATP] (Figure 1A right panel).^27^ Although theoretical studies indicate that the number and the size of substeps are not directly relevant to the chemo-mechanical coupling efficiency of F_1_,^28^ a clear negative correlation was found between the number of steps per turn in F_1_ and the number of steps per turn in F_o_ motor.^29^ This may provide some implications on the physiological demands or mechanistic constraints on the stepping behaviors of the F_1_ and F_o_ motors.

The crystal structures of F_1_ show that the γ subunit has principally two distinctive interactions with the α_3_β_3_ stator ring: the hydrophobic sleeve formed by the N-terminal domains of the α and β subunits, and the near-orifice region which is formed by the C-terminal domains of the β subunit.^4^^,^^30^ The hydrophobic sleeve is almost rotationally symmetric and has a smooth interface to the γ subunit that seemingly acts as an axis holder. In contrast, the near-orifice region shows a distinctively asymmetric interface. The three β subunits adopt different conformational states by swinging its C-terminal domain, forming the asymmetric interface with the γ subunit, which is believed to principally determine the rotary potential for the γ subunit in the α_3_β_3_-ring. The latest structural analysis of TF_1_^31^ revealed the atomic structure of F_1_ at the *binding dwell* state in which the γ subunit is at 40° in the rotational direction from the hydrolysis-waiting dwell structure. In these structures, two β subunits adopt new conformational states: half-closed and half-opened while the α subunits are in almost the same conformation as those found in previous structures of F_1_ in the *catalytic dwell* states. Thus, the structural features of F_1_ seemingly imply that the geometry of rotational potential for the γ subunit is dominantly determined by the β subunits.^32^

Considering the dominant role of the β subunits in the rotary potential and the catalysis, it is reasonable to consider that the fundamental features of F_1_ are principally determined by the β subunits. However, sequence homology analysis shows that the β subunit has the highest sequence identity among species (70% or more), whereas the α subunit is the second (60% or more) and the γ subunit is the lowest (40%) (Figure S2), implying that species-specific features stem more from the α and γ subunits with lower sequence identity. This raises a question regarding the design principle of F_1_, “which subunit or which combination of subunits is the most dominant for the rotary behaviors and kinetics?”.

In this study, we constructed several hybrid F_1_s composed of subunits from different origins, aiming to reveal the correlation between the origin of the subunit and the fundamental rotary properties of F_1_, namely, the rotational velocity and the number of steps per turn. As representatives of 3-stepper, 6-stepper, and 9-stepper motors, we employed PdF_1_, TF_1_, and *b*MF_1_, respectively, and prepared hybrid F_1_s composed of subunits from two of these origins (Figure 1B). The maximum rotational velocity of the hybrids was determined from the Michaelis-Menten analysis. On the other hand, the number of rotary steps per turn was analyzed as follows (Table S2). To resolve stepping rotation with discrete dwells, rotation was observed in the presence of ATPγS, an ATP analog that significantly retards the *catalytic dwell* and other dwells in some cases. To discriminate 3-stepper from 6- or 9-stepper motor, rotation was observed at [ATPγS] (or [ATP]) near $K_{m}$ where duration times of *binding* and *catalytic dwells* are comparable. Motors showing pauses for *binding* and *catalytic dwells* at the same angles at $K_{m}$ are identified as 3-stepper motors, while 6- or 9-stepper motors should show at least 6 pauses at $K_{m}$. To further discriminate between the 6- and 9-stepper motors, rotation was analyzed at ATPγS (or ATP)-saturating conditions where the 9-stepper motors show the extra pause, i.e., the *short dwell*.^26^ Finally, by structural comparison of the genuine F_1_s, the structural similarities among F_1_s that could affect the rotary properties of hybrid F_1_s are discussed.

## Results

### Preparation of hybrid F_1_

Hybrid F_1_s were prepared from expression plasmids in which one of the constituting genes was replaced with one from another origin as shown in Figure 1B. We introduce triplet characters in labeling the hybrids to indicate the origins (*P*, *T*, and *b* for PdF_1_, TF_1_, and *b*MF_1_, respectively) of the three subunits, α, β, and γ, e.g., “*bb*T” represents the hybrid with α-β from *b*MF_1_ and γ from TF_1_. We constructed 12 combinations of hybrids from two origins among the possible 18 combinations. Although T*bb* and PP*b* were not successfully expressed, the hybrids of other 10 combinations were obtained as F_1_ complexes. Among them, T*b*T and PP*b* did not show detectable ATP hydrolysis activity and did not show rotation (Table S3). In the following, we investigate 8 hybrids with the three original F_1_s for comparison. Note that SDS-PAGE analysis shows that PTT and TT*b* have apparently lower stoichiometry of the γ subunit, compared with other F_1_s, suggesting that the samples contained significant fraction of the α_3_β_3_ subcomplex or α/β monomers. However, this does not interfere rotation assay that selectively observes rotation via a probe attached to the γ subunit. The lower stoichiometry is probably due to lower expression, instability, or less efficient incorporation of the γ subunit into the α_3_β_3_ subcomplex. It should also be noted that the hybrid F_1_s were co-expressed with the δ and ε subunits originated from PdF_1_ or *b*MF_1_, to enhance the expression yield and stability. These minor subunits were incorporated in hybrid F_1_ complexes only when the γ subunit has the same origin. Exceptions were PPT and PTT, in which the ε subunit from PdF_1_ was incorporated in the hybrids, suggesting the association with the γ subunit from TF_1_. It should be noted that the minor subunits from PdF_1_ or *b*MF_1_ do not significantly affect rotation in catalysis of ATP hydrolysis,^10^^,^^11^^,^^13^ although the ε subunits from some of other bacterial F_1_s including TF_1_ is known to inhibit ATP hydrolysis and enhance the efficiency of ATP synthesis.^33^^,^^34^ Because the ε subunit from such F_1_s was not used in the present study, the hybrids were principally analyzed based on their composition of α, β, and γ subunits.

The rotation of hybrid F_1_s was visualized using a laser-dark field imaging system with 40 nm gold nanoparticle as rotary marker (Figure S4) and recorded at 250-10k fps (frames per second) as in previous studies.^26^^,^^27^ The rotation rate was determined under various [ATP] by Michaelis-Menten kinetics analysis (Figures S5A and S5B). The $V_{\max}$ and $K_{m}$ values of genuine F_1_s are found as follows: 786 rps (rotations per second) and 67 μM for *b*MF_1_, 338 rps and 77 μM for PdF_1_, 189 rps and 17 μM for TF_1_. These values largely agree with previous studies.^9^^,^^26^^,^^27^ The Michaelis-Menten parameters of the 8 hybrids are shown in Table 1. As expected, hybrids whose β originated from *b*MF_1_ or PdF_1_ showed fast rotation over 200 rps, whereas hybrids with β from TF_1_ showed slow rotation. In the following, we analyze which subunit is the dominant factor for $V_{\max}$. On the other hand, the catalytic efficiencies $k_{on}$, estimated from $3\times V_{\max}/K_{m}$, (Table 1) did not show significant differences among F_1_s. Thereby, these were not subject to further investigation.

**Table 1:** Subunit compositions and Michaelis-Menten parameters and $k_{on}^{ATP}$ values of the genuine F_1_s and hybrid F_1_s (n = 5) in ATP-driven rotation

	Name	α	β	γ	$V_{\max}$ (rps)	$K_{m}$ (μM)	$k_{on}^{ATP}$ (10^7^ M^−1^ s^−1^)
Genuine	*b*MF_1_	*b*	*b*	*b*	786 ± 4	67 ± 1	3.5 ± 0.2
	TF_1_	T	T	T	189 ± 2	17 ± 1	3.4 ± 0.3
	PdF_1_	P	P	P	338 ± 5^a^	77 ± 4^a^	1.3 ± 0.1^a^
Hybrid	*bb*T	*b*	*b*	T	608 ± 3	61 ± 2	3.0 ± 0.2
	P*b*P	P	*b*	P	477 ± 21	117 ± 19	1.2 ± 0.3
	PPT	P	P	T	228 ± 4	59 ± 5	1.2 ± 0.2
	PTP	P	T	P	123 ± 1	33 ± 2	1.1 ± 0.1
	TT*b*	T	T	*b*	57 ± 3	8 ± 2	2.2 ± 0.4
	*b*T*b*	*b*	T	*b*	17 ± 1	2 ± 0	3.0 ± 0.4
	PTT	P	T	T	9 ± 1	4 ± 2	0.7 ± 0.3
	*b*TT	*b*	T	T	5 ± 0	1 ± 0	2.0 ± 0.4

”*b*”, “T”, and “Pd” represent *b*MF_1_ origin, TF_1_ origin, and PdF_1_ origin, respectively. The catalytic efficiency $k_{on}^{ATP}$ was estimated from $3\times V_{\max}/K_{m}$. Errors represent fitting errors.

a Zarco-Zavala et al. 2020.

### Quadratic model

As shown in Table 1, $V_{\max}$ values differed among F_1_s, with no clear dependence on subunit origin. Nevertheless, we employed a simple quadratic model as a generic function with three variables that represent the origins of each subunit constituting hybrid F_1_s, as below;

$$V_{\max}\left({V_{\alpha },V}_{\beta },V_{\gamma }\right)=a_{\alpha }V_{\alpha }+a_{\beta }V_{\beta }+a_{\gamma }V_{\gamma }+a_{\alpha \beta }V_{\alpha }V_{\beta }+a_{\alpha \gamma }V_{\alpha }V_{\gamma }+a_{\beta \gamma }V_{\beta }V_{\gamma }+a_{\alpha \alpha }V_{\alpha }^{2}+a_{\beta \beta }V_{\beta }^{2}+a_{\gamma \gamma }V_{\gamma }^{2}$$

where $V_{\alpha }$, $V_{\beta }$, and $V_{\gamma }$ denote the explanatory variables for the α, β, and γ subunits, respectively. $V_{\alpha }$, $V_{\beta }$, and $V_{\gamma }$ can take one of the three values, $189rps∕\underline{V}$, $338rps∕\underline{V}$ or $786rps∕\underline{V}$, if the corresponding subunit is from the TF_1_, PdF_1_, or *b*MF_1_. The division by the average rotation rate, $\underline{V}=\frac{\left(189+338+786\right)}{3}\approx 438\left(rps\right)$, is to render the explanatory variables and the coefficients of the model dimensionless. For example, the hybrid P*b*P, with both the α and γ subunits from PdF_1_ and the β subunit from *b*MF_1_, has $V_{\alpha }=V_{\gamma }=338\left(rps\right)∕\underline{V}$ and $V_{\beta }=786\left(rps\right)∕\underline{V}$. The 9 coefficients $a_{\alpha },a_{\beta },a_{\gamma },\cdots ,a_{\gamma \gamma }$ characterizing both the linear and coupling strengths of the subunits on the rotation rate are least-squared fitted by the 11 genuine and hybrid F_1_s in Table 1. Their fitted values are summarized in Table 2. The quadratic model fits the data well with very small fitting residuals (defined as the difference between the observed and predicted $V_{\max}$ (see supplemental information “quadratic model of the dependence of rotation rate on subunits” and Figure S6). We also note that a model with any single term deleted from $V_{\max}\left({V_{\alpha },V}_{\beta },V_{\gamma }\right)$ increases the fitting residuals significantly, indicating that our quadratic model is minimal in explaining the data.

**Table 2:** Fitted coefficients of the quadratic model for $V_{\max}$ analysis

$\begin{array}{c} V_{\max}\left({V_{\alpha },V}_{\beta },V_{\gamma }\right)\\ {=a}_{\alpha }V_{\alpha }+a_{\beta }V_{\beta }+a_{\gamma }V_{\gamma }+a_{\alpha \beta }V_{\alpha }V_{\beta }+a_{\alpha \gamma }V_{\alpha }V_{\gamma }+a_{\beta \gamma }V_{\beta }V_{\gamma }+a_{\alpha \alpha }V_{\alpha }^{2}+a_{\beta \beta }V_{\beta }^{2}+a_{\gamma \gamma }V_{\gamma }^{2} \end{array}$	
Coefficients	Fitted value
$a_{\alpha }$	−2.508 ± 0.083
$a_{\beta }$	2.008 ± 0.070
$a_{\gamma }$	1.513 ± 0.073
$a_{\alpha \beta }$	0.469 ± 0.015
$a_{\alpha \gamma }$	0.179 ± 0.011
$a_{\beta \gamma }$	0.203 ± 0.013
$a_{\alpha \alpha }$	0.861 ± 0.036
$a_{\beta \beta }$	−0.865 ± 0.029
$a_{\gamma \gamma }$	−0.854 ± 0.035

Errors represent standard errors.

**Figure 2:**
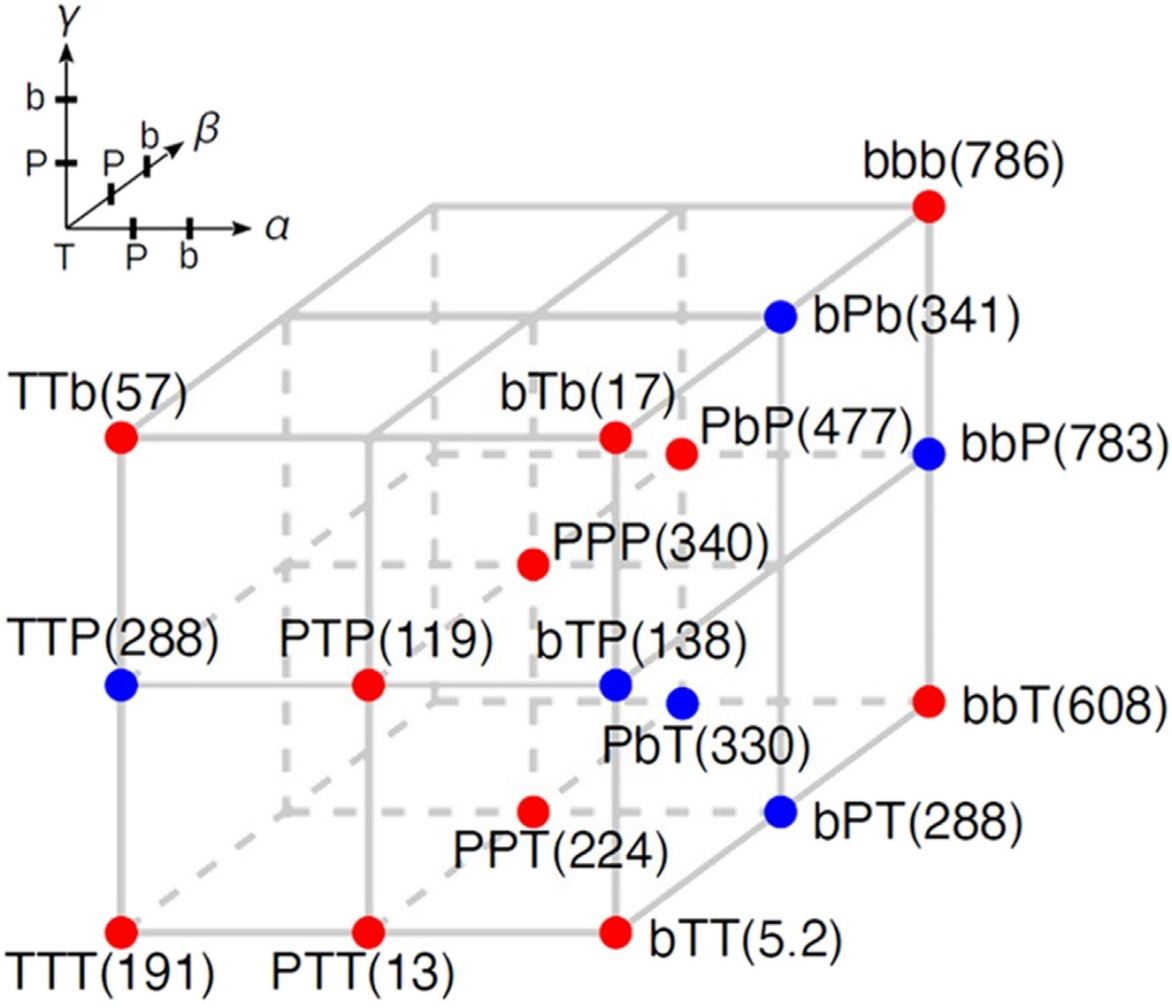
A lattice representation of $V_{\max}$ predicted by the quadratic model for the observed (red dots) and the unobserved hybrids (blue dots) The numbers (unit = rps) inside the parenthesis are the predicted value of $V_{\max}$ from the model. For the observed hybrids, the predicted values agree well with the observed $V_{\max}$ shown in Table 1. Prediction for unobserved hybrids (blue dots) is only performed for lattice points surrounded by observed values (red dots), i.e., interpolation. Predicting lattice points not surrounded by red dots corresponding to extrapolation is avoided since large bias can be resulted from extrapolation with polynomials.

A lattice representation of the predicted $V_{\max}$ for the observed hybrids (red dots) and some of the unobserved ones (blue dots) is shown in Figure 2. From the amount of changes of $V_{\max}$ in Figure 2 along the α-, β- and γ-axis, one can see that the β subunit has a dominant role in controlling the rotational velocity. Specifically, the change of $V_{\max}$ along the β-axis (see e.g., *b*TT→*b*PT→*bb*T that varies from 5.2 rps to 608 rps) is often much larger than those along the α-axis (see *e.g.*, TTP→PTP→*b*TP that varies from 288 rps to 138 rps) and along the γ-axis (see e.g., *bb*T→*bb*P→*bbb* that varies from 608 rps to 786 rps). These findings are consistent with the previous structural analysis^32^ suggesting the dominant roles played by the β subunit in the rotary potential and catalysis.

In addition to the range of $V_{\max}$ that can vary when a subunit with different origins is altered, the fitted coefficients (Table 2) also summarize the nonlinear dependence of $V_{\max}$ on the composition of the subunits. In particular, $a_{\alpha \alpha }>0$ means that $V_{\max}$ is a convex function of $V_{\alpha }$ when $V_{\beta }$ and $V_{\gamma }$ are fixed, i.e., the curve of $V_{\max}$ bends upward when the α subunit changes from TF_1_→PdF_1_→*b*MF_1_ with the β and γ subunits fixed (see e.g., TTT→PTT→*b*TT and TTP→PTP→*b*TP along the α-axis in Figure 2). Likewise, $a_{\beta \beta }<0$ $\left(a_{\gamma \gamma }<0\right)$ indicates that the curve of $V_{\max}$ is concave (bends downward) in $V_{\beta }$ $\left(V_{\gamma }\right)$ when $V_{\alpha }$ and $V_{\gamma }$ ($V_{\alpha }$ and $V_{\beta }$) are fixed (see *e.g.,* PTP→PPP→P*b*P and PTT→PPT→P*b*T along the β-axis in Figure 2).

On the other hand, the quadratic model characterizes the coupling effects between subunits. More precisely, the coefficients $a_{\alpha \beta }$, $a_{\beta \gamma }$, and $a_{\alpha \gamma }$ are all positive, meaning that having faster F_1_ parts in two of the subunits, i.e., αβ, βγ, or αγ, always increases $V_{\max}$. Furthermore, the fact that $a_{\alpha \beta }$ is around 2 to 3 times larger than $a_{\beta \gamma }$ and $a_{\alpha \gamma }$ (Table 2) implies that having faster F_1_ parts in the αβ subunits simultaneously enhances $V_{\max}$ the most. As a result, our results show that, in addition to the importance of the β subunit, the αβ subunits together further provide a synergistic effect controlling of $V_{\max}$. Accordingly, our model predicts that the unobserved hybrid *bb*P (see Figure 2) should have large *V*_max_ since both of its α and β subunits are derived from the fast-rotating *b*MF_1_ (due to the large $a_{\alpha \beta }$).

### Number of pauses

The number of steps per turn of the hybrids was then analyzed in order to classify the hybrids into 3-steppers (PdF_1_-type), 6-steppers (TF_1_-type), or 9-steppers (*b*MF_1_-type). 3-steppers showing 3 steps per turn regardless of [ATP]s have their *binding* and *catalytic dwells* at the same angles. On the other hand, 6-steppers and 9-steppers have their *binding* and *catalytic dwells* at different angles: the *catalytic dwells* are at 80^o^ in the counterclockwise direction from the *binding dwells*. When [ATP] is near $K_{m}$, rotation with 6 distinctive steps can be observed since the mean durations for the *binding* and *catalytic dwells* are comparable. Moreover, when the *catalytic dwells* are extended using ATPγS that is hydrolyzed at a slow rate, the rotary traces of the 6-steppers and 9-steppers show 6 well-resolved steps. The third pause of the 9-steppers, termed “*short dwell*”, is significantly short-lived compared with the *catalytic dwell*, and practically not observable when [ATP] is near $K_{m}$.

**Figure 3:**
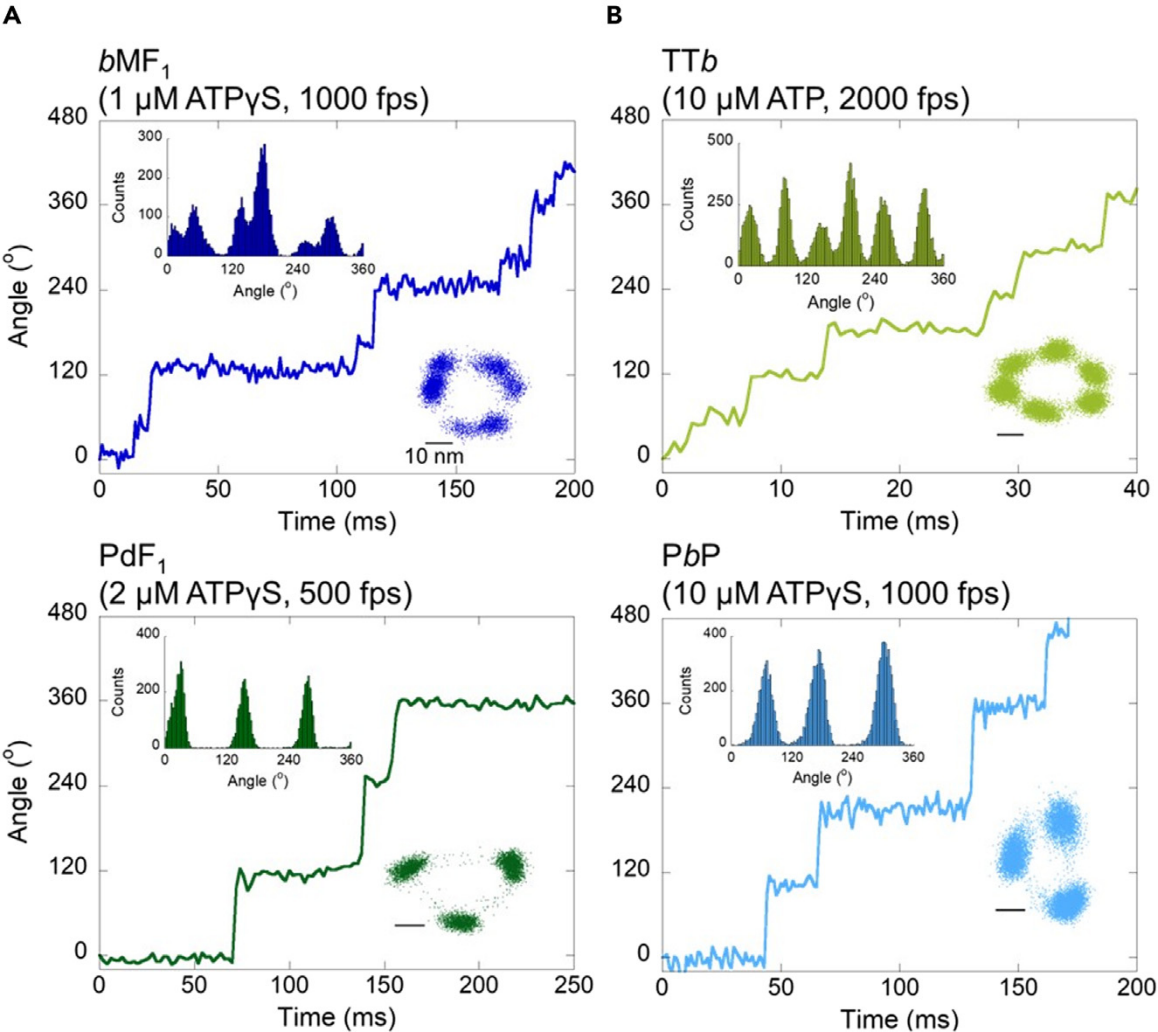
Rotation trajectories of (A) genuine and (B) hybrid F_1_s at [ATP] or [ATPγS] ≈ $K_{m}$ (*Upper inset*) histograms of angular positions (Bin width = 3^o^). (*Lower inset*) *x-y* plot. Experimental condition and frame rate are described in the figure. Other datasets are summarized in Figure S8.

We first distinguish the 3-stepper hybrids from the others in the rotary assay with [ATPγS] around $K_{m}$ (Figure S7A). For hybrids with slow catalytic rates, ATP was used as a substrate in the rotary assay. Michaelis-Menten parameters of the ATP- or ATPγS-driven rotation of hybrids were determined in the rotary assay and listed in Tables 1 and S4, respectively. Figure 3A shows the *x-y* plots and angular histograms of rotation of *b*MF_1_ or PdF_1_ as references for the 6-stepper or 3-stepper at [ATPγS] around $K_{m}$. All 8 hybrids clearly showed 6 or 3 pauses per turn, allowing for classification of the hybrids. Figure 3B shows the *x-y* plots and angular histograms of TT*b* and P*b*P as representatives of the 6-steppers and 3-steppers at [ATP] or [ATPγS] around $K_{m}$ (Tables 1 and S4). Other hybrids can also be identified as 6/9-stepper or 3-stepper (Figure S8). Surprisingly, all tested hybrids having a subunit with PdF_1_ origin (P*b*P, PTP, PPT, and PTT) showed 3 stepping rotations. This goes against our initial expectation that these hybrids showed 3 steps even if the β subunit is not PdF_1_ origin. A significant example is PTT that was identified as a 3-stepper although only the α subunit has PdF_1_ origin, probably suggesting the specific role of PdF_1_-α in controlling the stepping pattern. On the other hand, hybrids from the 6- and 9-stepper motors, *bb*T, *b*T*b*, TT*b*, and *b*TT, showed 6 steps at the $K_{m}$ condition without exception (Figures 3 and S8). The results of Pd-hybrid F_1_s (PPT, PTP, PTT, and P*b*P) suggested that the substep is not programmed in a particular subunit but in all of the subunits. Nevertheless, subunits with PdF_1_ origin have a dominant effect over other origins to determine the stepping behaviors of hybrid motors.

Next, we classify the non-3-stepper hybrids, which are composed of subunits with *b*MF_1_- and TF_1_-origins, into 6-steppers or 9-steppers (Figure S7B). As mentioned previously, the principal barometer is the existence of *short dwells* that are found in the rotation of *b*MF_1_. The difficulty is that these *short dwells* are very short-lived; the mean duration of *short dwells* of *b*MF_1_ is estimated to be less than 0.1 ms in ATP-driven rotation. These *short dwells* are extended to around 1.0 ms when *b*MF_1_ hydrolyzes ATPγS.^26^ For reliable classification, the hybrids of *b*MF_1_ and TF_1_, as well as the genuine *b*MF_1_ and TF_1_, were investigated under the substrate (ATP or ATPγS) saturating conditions. To objectively detect *short dwells* without the need to assume any noise models, we employed a nonparametric change-point (CP) analysis^35^ to identify the beginning and the end of rotary pause as “change-points” in the time-course of angular position of rotary probe. Based on quantitative statistical consideration (see supplemental information “change-point analysis”), CP analysis objectively and consistently identified the time instants at which the rotary angle changes abruptly with time resolution as fast as the recorded rates of the rotary trace, *i.e.*, no binning of the rotary trace was needed. The method has already been applied to identify angular changes in the single F_1_ rotary traces.^26^^,^^35^ The detection of the number of pauses was then performed by identifying the number of significant peaks in the angular histogram of the CP intervals without the need to refer to the time constants (see supplemental information “determining the number of pauses”). Note that CP analysis was introduced into this study to classify *b*M-T Hybrids into 9-stepper (*b*MF_1_-type) or 6-stepper type (TF_1_-type) by the number of pauses under substrate saturated-condition. The classification is based on the hypothesis that under substrate saturated-condition, 9-stepper F_1_s show 6 steps whereas 6-stepper F_1_s show only 3 steps due to overlapping of rotation phase of *long dwells* and *short dwells*.

**Figure 4:**
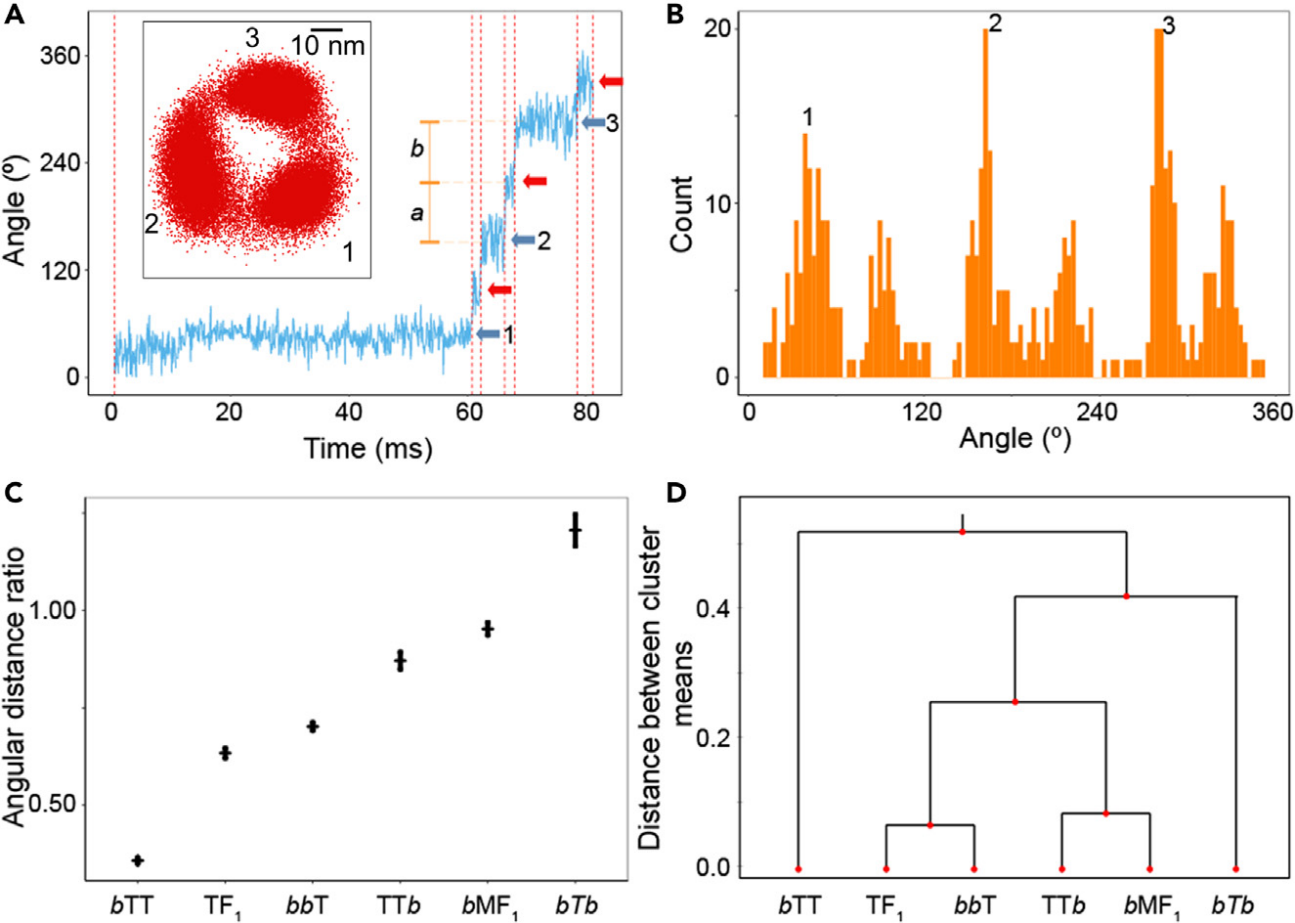
Determination of number of the pauses under 1 mM [ATP] or 1 mM [ATPγS] recorded at 10,000 fps (A) Example of rotation trace of *bb*T. (*Inset*) *x-y* plot. Red vertical dashed lines denote detected CPs. Blue and red arrows show the main and sub-pauses, respectively. “*a*” denotes the distance between mean angles of a main pause and the following sub-pause, and “*b*” denotes the distance between the mean angles of the same sub-pause and the following main pause. (B) Angular histogram of dwells detected by CP analysis in (A). The count (y axis) corresponds to the number of CP intervals, instead of the number of time points, where the angle (x axis) denotes the median angle of a CP interval. This way to construct the histogram is able to reveal the existence of short dwells in the rotary traces.^26^ (C) The mean of angular distance ratios a/b between main and sub-pauses for each hybrid. Short horizontal and vertical lines show the mean values and the standard errors of angular distance ratios after the outliers were removed. The number of sampling steps for the estimation of angular distance ratios is given in Table S5. (D) Hierarchical clustering (dendrogram) of the 6- and 9-steppers according to the angular distance ratio.

Consistent with the previous study,^26^ CP analysis successfully detected the *short dwells* of *b*MF_1_ around the middle in between two successive *catalytic dwells* (Figure S9). The histograms of other hybrids, as well as *b*MF_1_ and TF_1_ are provided in Figures S10 and S11. Figure 4A shows a sample segment of rotary trace of *bb*T at saturated [ATPγS] condition with the identified dwells detected by the CP analysis. Figure 4B shows the histogram of CP from the rotation of a *bb*T molecule, showing the distinctive 3 major and 3 minor peaks. Even though TF_1_ was expected to show only 3 *catalytic dwells*, TF_1_ showed 3 minor peaks, i.e*.*, 3 extra short pauses at binding angles, besides the *catalytic dwells* (Figure S10). It is known that TF_1_ conducts ATP binding and temperature-sensitive (TS) reaction at binding angles. According to our previous study,^36^ the mean duration time for TS pause is around 0.6 ms, significantly longer than the expected time constant of substrate binding at 1 mM, (<0.1 ms). Thus, it is most likely that the detected extra short pauses are TS reaction dwells. Supporting this contention, when the rotation of TF_1_ was observed at 1 mM [ATPγS] at 16°C, the pause time was extended to give a *Q*_10_ factor of 8.0 (Figure S12) that is close to the reported value for *Q*_10_ factor of TS reaction (7.0). The reason why TS dwell is detected in the present study is that CP analysis is sufficiently sensitive to detect short pauses that have not been identified with conventional angle histogram analysis or manually screening with eye.

In addition to TF_1_, CP analysis shows that all hybrid F_1_s that do not involve a subunit with PdF_1_ origin showed 3 extra minor dwells in between the major ones (Figure S10). In some hybrids, the 3 extra minor dwells were not clear in conventional angle histogram. This is due to the same reason for the detection of TS dwells in TF_1_. Next, we investigate the position of the extra short pauses relative to the major ones by analyzing the angular distance ratio, defined as *a/b* in Figure 4A where *a* (*b*) is the angular distance from a main (sub-) pause to the next sub- (main) pause (see supplemental information “step size comparison”). In this analysis, the *b*MF_1_ gives the angular distance ratio close to 1.0 (60^o^:60^o^) while the TF_1_ gives the ratio close to 0.5 (40^o^:80^o^) (Table S5). Figure 4C shows the average values of the angular distance ratios determined for the hybrid F_1_s, *b*MF_1_, and TF_1_. The ratios for the hybrids do not show simple classification into the *b*MF_1_-type or TF_1_-type. To gain more insights, we investigated the similarities between all 6- and 9-steppers by hierarchical clustering according to the mean of the angular distance ratios. Figure 4D shows the resultant dendrogram representing the hierarchical similarities among the F_1_s. The dendrogram suggests that TF_1_ and *bb*T are similar to each other, whereas *b*MF_1_ and TT*b* form another group, and these four form a group with *b*T*b* later which means less similarity with them whereas *b*TT stands alone by itself. These groupings suggest that a simple and explicit rule on which factor determines the number of rotary pauses may not be present. However, the analysis indicates that the angular distance ratio is larger when the γ subunit originates from *b*MF_1_ than those with TF_1_ origin.

## Discussion

### High robustness against interspecies subunit substitution

In this study, we constructed the expression vectors for 12 hybrid F_1_s. Eight out of the 12 hybrids form F_1_ complexes that exhibit active catalysis and rotation (Tables S3 and S6). Considering the separation of the 3 genuine F_1_s (TF_1_, PdF_1_, and *b*MF_1_) in phylogenetic trees, the high success rate of forming active hybrids indicates that the subunit-subunit interfaces are structurally conserved. The highly permissive recognition by the α_3_β_3_ subcomplex to accommodate an exogenous rotor protein in the α_3_β_3_ ring was reported in previous studies. Grüber et al. succeeded in construction of an artificial protein from α_3_β_3_ of TF_1_ and a rotor subunit E of V_1_-ATPase from yeast, which showed 56% of the ATPase activity of the genuine F_1_.^37^ We also demonstrated in a previous study that it is possible to substitute the γ subunit with a completely exogenous rod protein FliJ of the *S. enterica* flagella.^38^ The rotary catalysis of that artificial motor protein was experimentally confirmed although the artificial motor showed evidently slow rotation rate, exerting low torque. These results show the structural and functional robustness of the α_3_β_3_ subcomplex against exogenous rotor protein. The high success rate to obtain active hybrid F_1_s with the γ subunit from a different species is consistent with the previous reports.

An unexpected result to us was the robustness of F_1_ against interspecies substitution of the α and β subunits. One reasonable expectation is that although the evolutional constrain on the α and the β subunits is high to retain the fundamental rotational catalytic mechanism, the molecular mechanisms for allosteric interplay among subunits and torque transmission have been diversified in course of evolution, as seen in the diversification of the number of substeps. The observed robustness implies that the fundamental molecular architecture for the allosteric interplay and torque transmission among subunits are more highly conserved than we thought. The higher conservation of amino acid sequence of the α and β subunits would reflect this.

The unraveled robustness of F_1_ against interspecies substitution would open a novel experimental strategy for the study on F_1_s that are experimentally difficult to obtain for biochemical/biophysical studies, such as F_1_s derived from species that are difficult to culture and/or have low structural stability. Recent innovation in structural analysis of F_o_F_1_ has been revealing that F_o_F_1_ has wide variety of regulation mechanisms, evolving species-specific regulatory subunit or structural elements.^13^^,^^39^ However, the types of F_1_ suitable for biochemical and biophysical analysis are still limited. In many cases, species-specific regulation mechanisms are based only a few subunits or structural elements. By introducing the necessary subunits and structural elements into F_1_ suitable for biochemical/biophysical research, it will be possible to create easy-to-handle hybrid F_1_ with the species-specific regulation mechanism. This will enable detailed biochemical/biophysical analyses, which have been difficult with native F_1_.

### Rotation rate

In terms of the lattice representation (Figure 2), we showed the maximum turnover rates $V_{\max}$ of the hybrid F_1_s determined from the Michaelis-Menten analysis and predicted from the quadratic model. The $V_{\max}$ values vary the most with respect to the exchange of β subunits, showing that the β subunit is a determining factor of $V_{\max}$. We further scrutinize how the subunit dependences of $V_{\max}$ are characterized by the quadratic model. In particular, the coefficients for the subunit combinations ($a_{\alpha \beta }$, $a_{\beta \gamma }$, and $a_{\alpha \gamma }$) are all positive with $a_{\alpha \beta }$ the biggest, indicating that the α and β subunits together provide a synergistic effect on the rotational velocity. This synergistic effect of α and β subunits is consistent with the fact that the catalytic sites reside on the α-β interfaces, and with the crucial roles of their conformational transitions associated with the hydrolysis and phosphate releasing steps that determine the catalytic rate.

The rate of $V_{\max}$ is set by the duration time of *catalytic dwell*. The structural feature of *catalytic dwell* state is well represented by the ground state structures of *b*MF_1_ in which β_DP_ represents catalytically active form on which bound ATP is cleaved into ADP and P_i_. While the conformation of the β subunit itself is almost identical to that of β_TP_, the αβ_DP_ interface is more closed than the αβ_TP_ interface. The closure of αβ_DP_ interface accompanies the positional shift of the arginine finger on the α subunit toward the bound ATP that is thought to trigger the hydrolysis of bound ATP.^8^^,^^9^ Therefore, it would be natural to expect that the difference in $V_{\max}$ is represented in the structural arrangement of catalytic residues including arginine finger on αβ_DP_. The atomic structure model of PdF_1_ and TF_1_ in *catalytic dwell* state as well as *b*MF_1_ are now available.^31^^,^^40^ To investigate possible conformational differences of the catalytic residues among the 3 genuine F_1_s, we investigated the structural similarity of catalytic residues by performing structural alignment (Figure S14). However, the atomic coordinates of catalytic residues are so identical that we observed negligibly small RMSD (root-mean-square deviation), around 0.1 nm. Thus, the coordinates of catalytic residues do not provide clues on which structural feature determines $V_{\max}$. Based on QM/MM (Quantum Mechanics/Molecular Mechanics) study, Hayashi *et al.* showed that post-hydrolysis proton transfer process mediated by water molecules coordinated in catalytic site is the rate-limiting step of overall hydrolysis step process that was verified with rotation experiments.^9^ Taking this into account, it is possible that the coordinates of water molecules and/or those of residues involved in water molecule coordination determine $V_{\max}$.

In addition to hydrolysis step, the P_i_ release step is also another determining factor of duration time of *catalytic dwell*. It was proposed that the α-β interface loosens to facilitate the phosphate release in the final stage of the ATP-hydrolysis.^32^^,^^35^ This contention is well consistent with the synergistic effect of α and β subunits for $V_{\max}$. Therefore, it is also possible that structural features involved in P_i_-releasing step determine the species-specific $V_{\max}$. Regarding this point, further structural studies are required because it remains elusive which type of conformational transition is accompanied with P_i_ release.

### Number of substeps

We also investigated the correlation between the origin of the subunit and the number of steps per turn of the F_1_s. TF_1_ makes 6 steps per turn, pausing at 0°, 80°, 120°, 200°, 240°, and 320°. Here, 0°, 120°, and 240° are the *binding dwells* and 80°, 200°, and 320° are the *catalytic dwells*. Recently, the structure of TF_1_ in the binding state was established in addition to that of the catalytic state.^31^ While the conformation of α and γ subunits does not significantly change in all the binding and catalytic states, the β subunit adopts distinctive conformational states at different angles: half-closed form (HC) at 0°, closed form (C) at 80°, 120°, and 200°, half-opened form (HO) at 240°, and opened form (O) at 320°. These structural features of the β subunit seemingly suggest the *β-dictator model* stating that the conformational substates of F_1_ found as intervening pauses are principally determined by the β subunit. However, the results in the present study do not support this model. Remarkable examples are the 3-stepper hybrids, P*b*P, PTP, and PTT, with the β subunit originated from TF_1_ (6-stepper) or *b*MF_1_ (9-stepper). These hybrids show that the incorporation of PdF_1_ subunit results in a 3-stepper motor even if the β subunit has a different origin. The reason for apparently PdF_1_-dictator mechanism remains elusive.

Similarly, the hybrids originated from TF_1_ and *b*MF_1_ are also inconsistent with the *β-dictator model*. The analysis of the angular distance ratios (Figure 4D) suggests that TF_1_ and *bb*T are similar to each other, while *b*MF_1_ and TT*b* form another group. *b*T*b* and *b*TT stand relatively alone by themselves. Although a universal rule is yet to be found, our results suggested that the γ and β subunits may have a larger effect on the number of substeps than the α subunit. Instead of the *β-dictator model*, our analysis shows that the number of steps per turn is determined not by a single factor, but by the nontrivial interplays among all subunits.

With aim to investigate which rotor-stator interface is responsible for the number of substeps, we compared the rotor-stator interfaces of 3 genuine F_1_s (Figure S15). The stator interface against the rotor can be categorized into hydrophobic sleeve, switch II, and DELSEED regions. The highest differences among the three species are found in DELSEED region; higher RMSD values for DELSEED interface, in comparison with those for switch II or hydrophobic sleeve. This result implies that DELSEED interface has a principal role to determine the number and size of substeps. Interestingly, amino acid sequences of interface between DELSEED and γ is highly identical among species. This suggests that the scaffold structures behind DELSEED region in addition to the rotor structure are responsible for the diversification of the rotation scheme of F_1_. This idea would be testable by “chimera approach” where a certain distinctive structural domain, such as C-terminal helical domain of the β subunit including DELSEED region, is substituted with the corresponding part from another origin. In particular, the analysis on F_1_ having a chimera subunit with PdF_1_ subunit could give more detailed insights on which structural element or which part of stator-rotor interfaces are responsible for the number and/or angles of substeps of F_1_.

### Conclusions

In summary, this study analyzed hybrid F _1_s which are composed of subunits with different origins (Table S6). Kinetic analysis of the hybrids revealed that the principal factors to determine the maximum turnover rate are the β subunit and the synergistic combination of α and β subunits, which is consistent with the structural features of F_1_.^32^^,^^41^^,^^42^ The step analysis of the hybrids showed that the number of steps per turn is not determined by a single factor but by nontrivial interplays among all subunits. For further understanding of how the number of steps and step-size are determined, detailed analyses of the kinetics and rotary behaviors are required. Structural analysis of the hybrid F_1_s could also give crucial clues on which structural features are responsible for the catalytic rate and the number of steps per turn. We also propose that our subunit exchange strategy would be effective not only for the understanding of the design principle of molecular motors, but also for the engineering of novel motors.

### Limitations of the study

In the present study, we focus on the rotary behaviors of hybrid F_1_s utilizing single-molecule rotation assays. To obtain further insight on the hybrid F_1_s, the structural analysis is required in future. In particular, the structural information of α_3_β_3_-ring and γ could provide us with more insights on the regions determining the *V*_max_ and the complicated interplays among all the subunits determining the number of pauses per turn. Nevertheless, our study can provide phenomenological and dynamical information for the rotary behaviors of F_1_s that complements comprehensive structural analyses. It also enables us to design novel hybrid F_1_s with predictable rotary behaviors by replacing the dominant regions among genuine F_1_s.

## STAR★Methods

### Key resources table

**Table:** 

REAGENT or RESOURCE	SOURCE	IDENTIFIER
**Bacterial and virus strains**		

HIT JM 109	Real Biotech Corporation	Cat#RH717
BL21(DE3) Δunc	Nichols et al.^43^	https://doi.org/10.1128/jb.179.16.5056-5061.1997
JM103 Δunc	Monticello et al.^44^	https://doi.org/10.1128/jb.174.10.3370-3376.1992
DK8	Klionsky et al.^45^	https://doi.org/10.1128/jb.160.3.1055-1060.1984

**Chemicals, peptides, and recombinant proteins**		

Gold nanoparticle (φ = 40 nm)	BBI solutions	CAT#SKU EM.GC40/7
ATP	Sigma-Aldrich	CAT# A7699
ATPγS	Roche	CAT# 11162306001
Phospho(enol)pyruvic acid monosodium salt hydrate	Sigma-Aldrich	P0564-1G
Pyruvate kinase from rabbit muscle	Roche	CAT#10109045001
Bovine Serum Albumin	Sigma-Aldrich	CAT#A3294-10G
Lauryl Dimethylamine Oxide (LDAO)	Sigma-Aldrich	CAT#40236-250ML

**Critical commercial assays**		

KOD -Plus- NEO	TOYOBO	CAT#KOD-401

**Oligonucleotides**		

Primer for preparation of hybrid F_1_s, see Tables S7 and S8	This paper	N/A

**Software and algorithms**		

custom software	Visual C# 2008	https://journals.aps.org/prl/abstract/10.1103/PhysRevLett.104.198103
quadratic model fitting, see supplemental information	This paper	N/A
change-point analysis algorithm, see supplemental information	Li, et al.^35^	https://www.nature.com/articles/ncomms10223
clean-up algorithm, see supplemental information	Kobayashi, et al.^26^	https://www.pnas.org/doi/abs/10.1073/pnas.1909407117
determining the number of pauses, see supplemental information	This paper	N/A
step size comparison, see supplemental information	This paper	N/A
Kaleida graph	HULINKS	https://www.synergy.com/

**Other**		

dark-field microscope	Ueno et al.^46^	https://www.sciencedirect.com/science/article/pii/S0006349510001384?via%3Dihub

### Resource availability

#### Lead contact

Further information and requests for resources and reagents should be directed to and will be fulfilled by the lead contact, Dr. Hiroyuki Noji (hnoji@g.ecc.u-tokyo.ac.jp)cbli@math.su.se.

#### Materials availability

The plasmids generated in this study are available upon reasonable request.

### Method details

#### Preparation of hybrid F_1_s

Genuine TF_1_ and *b*MF_1_ were prepared as described.^26^^,^^47^ As an example, a detailed process of the preparation of *bb*T is as follows. The plasmid of *bb*T was constructed from the *b*MF_1_ plasmid as a vector and PCR product coding TF_1_-γ as an insert DNA. The primers for PCR reactions are summarized in Table S7. The PCR fragment consists of TF_1_-γ and the upstream/downstream of *b*MF_1_-γ. After ligation of the vector and the fragment, the plasmid of *bb*T was introduced into the F_o_F_1_-deficient *Escherichia coli* strain, BL21. The protein purification of TF_1_-*b*MF_1_ hybrids were performed based on the purification method for *b*MF_1_.^26^ The hybrid F_1_s consisting of subunits from PdF_1_ were expressed and purified as described previously.^27^ PdF_1_ plasmid was used as a vector. The primers for PdF_1_-hybrids are summarized in Table S8. After purification of the target proteins, conformation of α_3_β_3_γ_1_ was confirmed with SDS-PAGE analysis. Note that the process of the size exclusion chromatography was omitted in the purification process of the samples of hybrid F_1_s consisting of subunits from PdF_1_ for rotation assay.

#### Single-molecule rotary assay

To visualize the rotation of F_1_s, two cysteine residues on the γ subunit of *b*MF_1_ (γA99C, γS191C), TF_1_ (γS109C, γI212C), and PdF_1_ (γQ115C, γD214C) were introduced and biotinylated to attach 40 nm diameter gold nanoparticles as an optical probe. The processes are as follows. The flow cell was constructed from 2 cover glasses (18 × 18 mm^2^ and 24 × 32 mm^2^; Matsunami Glass) using double-sided tape as a spacer. The surface of the bottom glass was coated with Ni-NTA. The basal buffer for the assay of *b*MF_1_ or TF_1_-*b*MF_1_ hybrid F_1_s contained 50 mM Hepes-KOH (pH 7.5), 50 mM KCl, and 3 mM MgCl_2_. The basal buffer for the assay of TF_1_ contained 50 mM MOPS-KOH (pH 7.0), 50 mM KCl, and 3 mM MgCl_2_. When ATP was used, an ATP-regenerating system (1 mM phosphoenolpyruvate and 50 μg/mL pyruvate kinase) was added to the basal buffer. First, the flow cell was incubated with BSA buffer, the basal buffer containing 5 mg/mL BSA, for 5 min. Next, F_1_ molecules of 0.3–10 nM in the BSA buffer were infused and incubated for 10 min. Then, unbound F_1_ molecules were washed out by the BSA buffer and 40 nm gold nanoparticles were infused and incubated for 10 min. Unbound particles were washed out with the basal buffer containing substrate. The rotary assay was conducted with a dark-field microscope^46^ (OLYMPUS IX-71) with a 60× objective lens at the recording rate of 250-10k fps (FASTCAM-1024PCI or FASTCAM NOVA s-16, Photron, Japan) (Figure S4). Recorded movies were processed by a custom software.^48^ The localization precision and signal-to-noise ratio ranges were the same as the previous studies.^26^^,^^46^ When it is hard to find rotating particles, the F_1_ concentration was increased up to 300 nM. When no rotating particle was observed analyzing typically over 1500 particles showing tethered rotary Brownian motion, we concluded that the hybrid F_1_ does not rotate. To determine the number of pauses, ATPγS was used as a substrate in the rotary assay of the hybrid F_1_s, except for whose $V_{\max}^{ATP\gamma S}$ values were more than 5 rps. For PdF_1_ and its hybrids, the procedures were performed as described in.^27^ Note that the basal buffer for PdF_1_ and Pd-hybrids contained 50 mM Tris-acetate (pH 8.0), 30 mM CH_3_COOK, and 250 mM Sucrose, and 0.1% LDAO (lauryldimethylamine oxide). Methods for the kinetic and statistical analysis of the stepping behavior are described in the Supplemental text.

#### Quadratic model of the dependence of rotation rate on subunits

The fitting of the quadratic model for the $V_{\max}$ values was performed by least square fitting. To confirm if all 9 terms, especially the nonlinear terms, in the quadratic model were needed, the fitting residuals were checked if any one of the terms was dropped. It was found that the fitting residuals increased significantly by dropping any one of the terms, indicating that our quadratic model is minimal in describing the observed rotation rates. On the other hand, including a constant term in the quadratic model resulted in an almost zero constant without visible improvement of the fitting residual, the constant term was therefore dropped in our quadratic model.

#### Change-point analysis

##### Detection of change-point (CP)

A nonparametric change-point (CP) analysis^26^ was used to detect changes in the traces. We wanted to find the points where a sudden change happened in the trace. The steps of CP analysis were as follows. A permutation test was used to check if there existed a CP in a segment of trace. In this test, the null hypothesis was that there was no CP and the alternative hypothesis was that there was at least one CP. As a test statistic, cumulative sum (CUSUM) was used, which was defined as $CUSUM\left(t\right)=\sum_{t^{\prime }=1}^{t}\left(x_{t^{\prime }}-x^{¯}\right)$ where t=1,···,n is time point for the segment $\left(x_{1},x_{2},\ldots ,x_{n}\right)$ and $\underline{x}$ is the mean angle of the segment. It is the sum of differences between the angles of points and the mean of the segment. This means that if there exists a change point, the fluctuation of CUSUM will be larger. Therefore, it is better to use D=(CUSUM(t))−min(CUSUM(t)) as a test statistic since it shows the total fluctuation of the CUSUM. Following the permutation test, if the null hypothesis is rejected, then the probable location of CP should be found. It is the point which has the smallest total squared error from fitted mean values of left and right segments of the point under consideration.

These steps were repeated for each segment to find multiple CPs in the trace. First, CP with the largest D was detected and segments were divided into two parts. Then the permutation test was applied again to new segments and they were divided if a new CP was detected. The process was repeated until no new CP could be detected.

Then, a histogram was constructed for CP intervals (Figure S9, *Second row, Left*). Mean angular value of each CP interval was counted only once for this histogram. Therefore, even short pauses can be observed in it.

##### Cleaning up procedure

Besides true change points, several extra CPs could be detected due to some undesired fluctuations in the trace. Thus, after the detection of CPs, a cleanup procedure was performed. For *i*-th CP interval with *n* data points, the median ${x_{i}}^{∼}$ and the median absolute deviation ${MAD}_{i}=median\left(\left|x_{1}-{x_{i}}^{∼}\right|,\ldots ,\left|x_{n}-{x_{i}}^{∼}\right|\right)$ were obtained. Mean and standard deviation were not used since they are not robust to outliers. Then, for every consecutive CP interval, we checked if the CP between them could be removed by comparing the difference of their median angles and the sum of their MADs multiplied by a constant A. If $\left|{x_{i}}^{∼}-{x_{i+1}}^{∼}\right|<A\times \left({MAD}_{i}+{MAD}_{i+1}\right)$, then CP between *i*-th and (*i*+1)-th CP intervals were removed. If a larger A was chosen, more CPs would be removed. Therefore, it is better to start with a small A. Then, the squared error between the trace before the cleanup procedure and the denoized trace was estimated. This process was repeated by increasing A, we obtained a plot of squared error and A factor (Figure S9, *First row, Right*). In this plot, there is a rapid increase in squared error as A increases. Before this rapid increase of the squared error, undesired CPs due to fluctuations are removed; however, after the rapid increase, the true CPs start to be removed. Therefore, a good choice of A factor is just before the sudden increase in the plot. After the cleanup procedure, the peaks in the histogram for CP intervals became sharper (Figure S9, *Second row, Right*).

#### Determining the number of pauses

After the CP analysis and the cleanup procedures, the histogram of CP intervals was constructed for each molecule of a given hybrid. In some cases, the number of peaks in the histogram may still not be easy to determine visually. Therefore, further clean-up procedure was implemented to sharpen the histogram. First, too short CP intervals, whose length was shorter than 7 points, were dropped. There also exists a few CP intervals corresponding to backward steps in the rotary trace or undesired small angular fluctuations from the measurement. To identify these undesired CP intervals, we compared the median angles of each CP interval to its previous and next CP intervals. If the difference between them was smaller than 40° and the medians do not progressively increase, the CP interval under consideration was removed from the histogram. Following these procedures, the visual detection of the number of peaks in the histogram became much easier. Two histograms for each hybrid are shown in Figure S10 for the 6- (or 9-) steppers and Figure S11 for the 3-steppers.

#### Step size comparison

After the number of peaks were determined, it can be seen that some molecules show 6 distinct pauses, suggesting that there is a sub-pause between each pair of main pauses. Boundaries were then placed between the pauses in the histogram (see *e.g.,* Figure S13) so that each CP interval can be uniquely assigned to one of the 6 pauses. We note that there exist some fluctuations in the angle separations between consecutive main pauses, *i.e.*, the separations do not exactly equal to 120^o^, due to, *e.g.*, the titling of the motor rotary plane relative to the imaging axis, etc. To take into account these fluctuations, we considered the ratio of the distance between median angles of a main pause and the following sub-pause (“*a*”, Figure S13), and the distance between the median angles of the same sub-pause and the following main pause (“*b*”, in Figure S13). The angular distance ratio is smaller when the sub-pause is closer to the main pause before that. Finally, hierarchical clustering was used to compare the mean angular distance ratios of six hybrids with 6 pauses (Figure 4D).

### Quantification and statistical analysis

All details regarding statistical analyses are described in the Methods, Figure legends, and supplemental information. Data analysis was performed using Kaleida graph (HULINKS) or Mathematica (WOLFRAM).

## Data Availability

All original code will be deposited at [https://github.com/rwatanabe0515/Hybrid_paper.git] and is publicly available as of the date of publication. DOIs are listed in the key resources table. The raw data reported in this paper have been deposited at Mendeley data, https://doi.org/10.17632/2hfsfk5zdj.1. Any additional information required to reanalyze the data reported in this paper is available from the lead contact upon reasonable request.
