# Supplementary material for: Rotary properties of hybrid F1-ATPases consisting of subunits from different species
Source: iScience. 2023 Apr 8;26(5):106626. doi: 10.1016/j.isci.2023.106626 (PMC10182284; doi:10.1016/j.isci.2023.106626)
Supplement: Document S1. Figures S1–S16 and Tables S1–S8 [file mmc1.pdf]

iScience, Volume *26*

## Supplemental information

# Rotary properties of hybrid $F_1$-ATPases consisting of subunits from different species

**Ryo R. Watanabe, Busra Tas Kiper, Mariel Zarco-Zavala, Mayu Hara, Ryohei Kobayashi, Hiroshi Ueno, José J. García-Trejo, Chun-Biu Li, and Hiroyuki Noji**

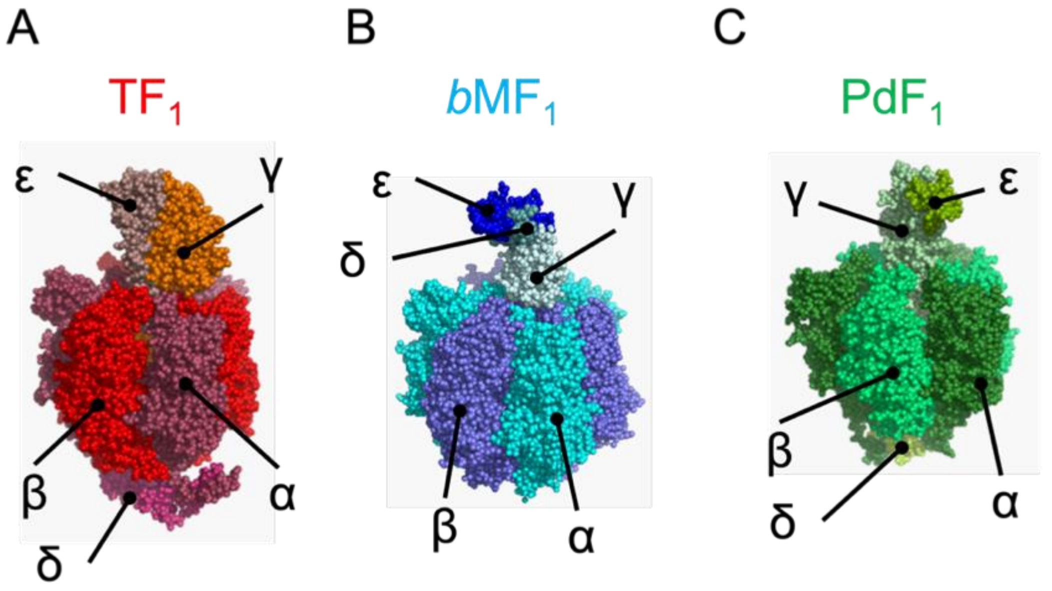


**Figure S1. – The subunit compositions of genuine $F_1$s of $TF_1$ (*A*), *b*$MF_1$ (*B*), and $PdF_1$ (*C*). PDB ID: 6N2Y as $TF_1$, 2JDI as *b*$MF_1$, and 5DN6 as $PdF_1$, related to Figure 1.**

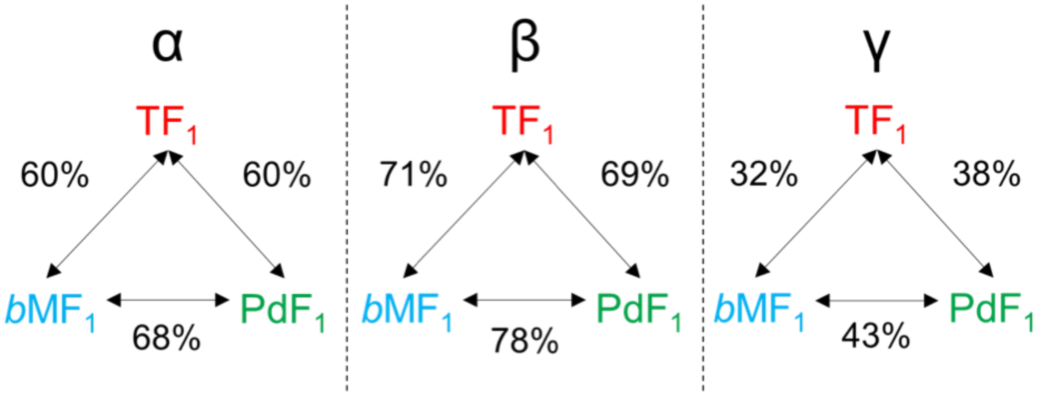


**Figure S2. – Identity of amino acid sequence of each subunit among species calculated by ClustalX (PDB ID: 1BMF as *b*MF$_1$, 4XD7 as TF$_1$ and 5DN6 as PdF$_1$), related to Figure 1.**

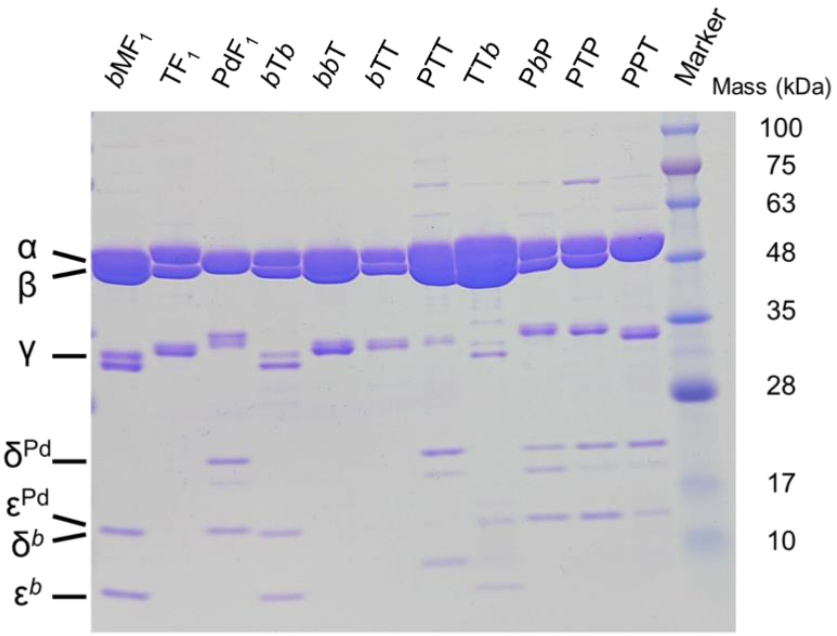


**Figure S3. – SDS-PAGE analysis of the purified hybrid $F_1$s, related to Figure 1 and STAR Methods.**

$F_1$s were analysed with 10 - 20% gradient gel (nacalai tesque) after biotinylation. $\delta^b$ and $\varepsilon^b$ represent the δ and ε subunits derived from *b*MF₁. $\delta^{Pd}$ and $\varepsilon^{Pd}$ represent the δ and ε subunits derived from $PdF_1$. In the most hybrids as well as genuine $F_1$s, the γ subunit showed doubled bands, due to partial biotinylation; higher band corresponds to fully biotinylation while lower is not fully biotinylated fraction. The effect of partial biotinylation on the doubled band is shown in Fig. S16.

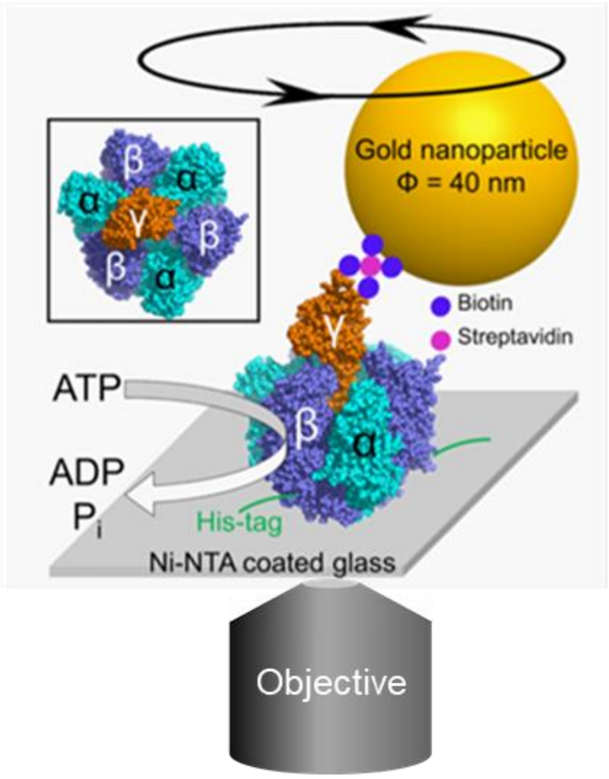


**Figure S4. – Experimental system of single-molecule observation of $F_1$s, related to STAR Methods.**

The $F_1$ molecule was attached on a surface of Ni-NTA coated glass by His-tags on βs. A gold nanoparticle (φ: 40 nm) was attached to the γ as a rotation probe via biotin-streptavidin interaction. The rotation of the probe was observed with total internal dark-field microscopy.

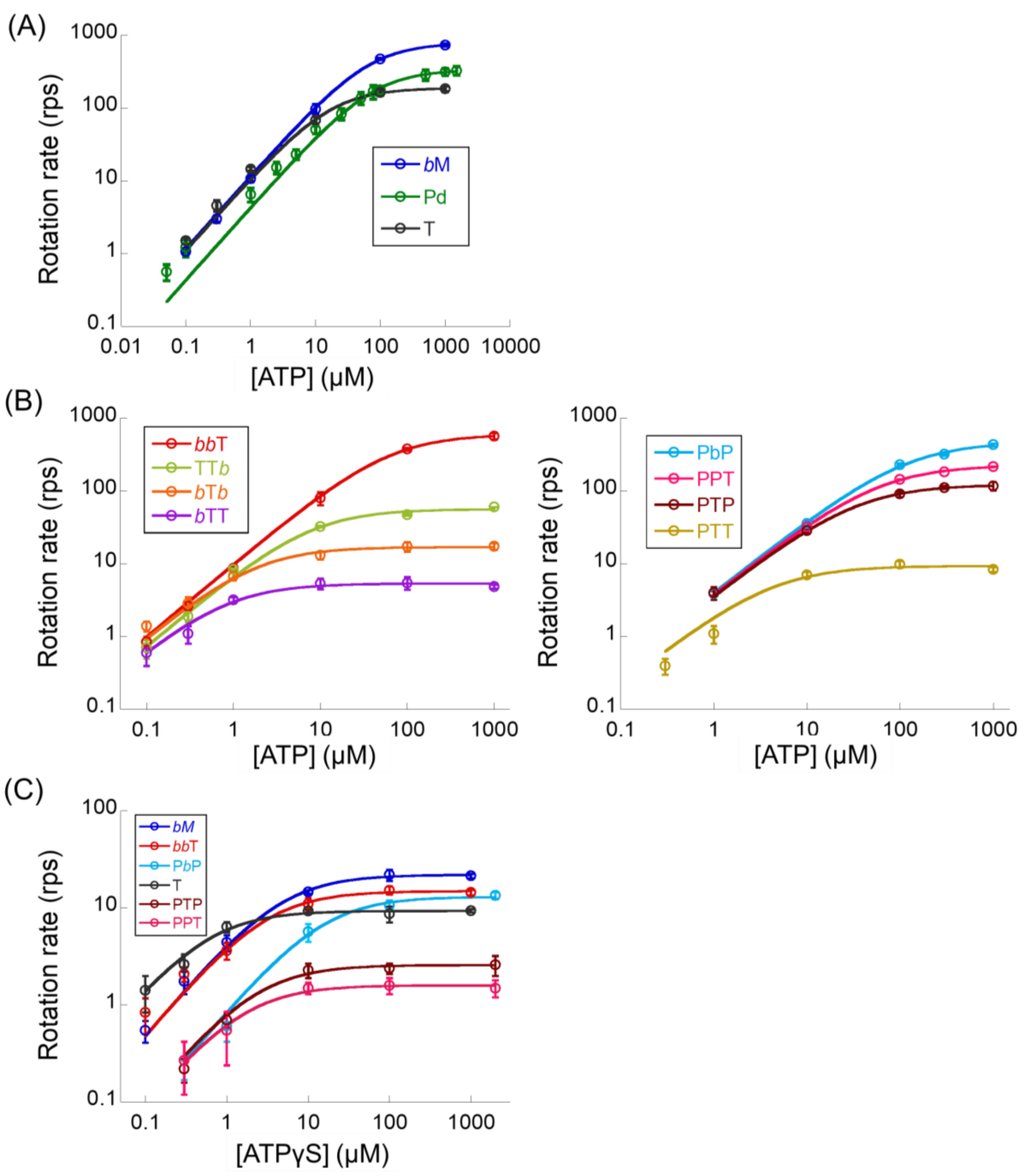


**Figure S5. – [ATP]-dependency of the rotation rate of $F_1$s (A) genuine $F_1$s, (B) hybrid $F_1$s, (C) [ATPγS]-dependency of the rotation rate of $F_1$s (*n* = 5), related to Table 1.**

The detail procedure of single-molecule rotary assay is described in the Method part. We measured the rotation rate of molecules which continuously rotate for more than 5 revolutions as criteria. The data were fitted by the single Michaelis-Menten equation. The values of $K_m$ and $V_{max}$ are shown in Tables S4 and S6. Errors represent standard deviations.

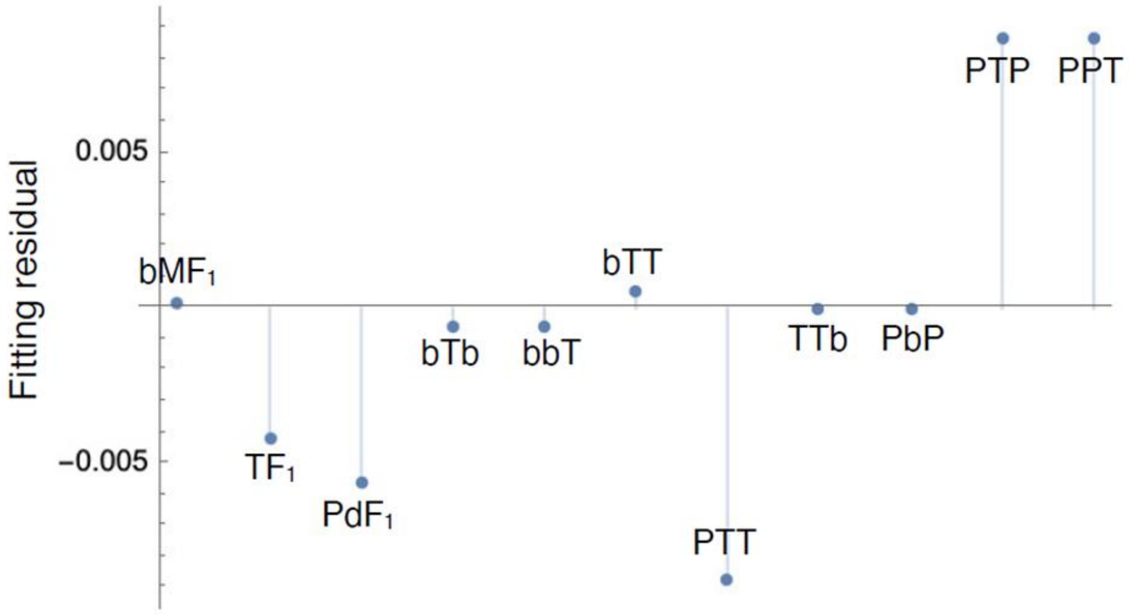


**Figure S6. – Fitting residual of the $V_{max}$ value of each $F_1$ in the quadratic model, related to Figure 2 and Table 2.**

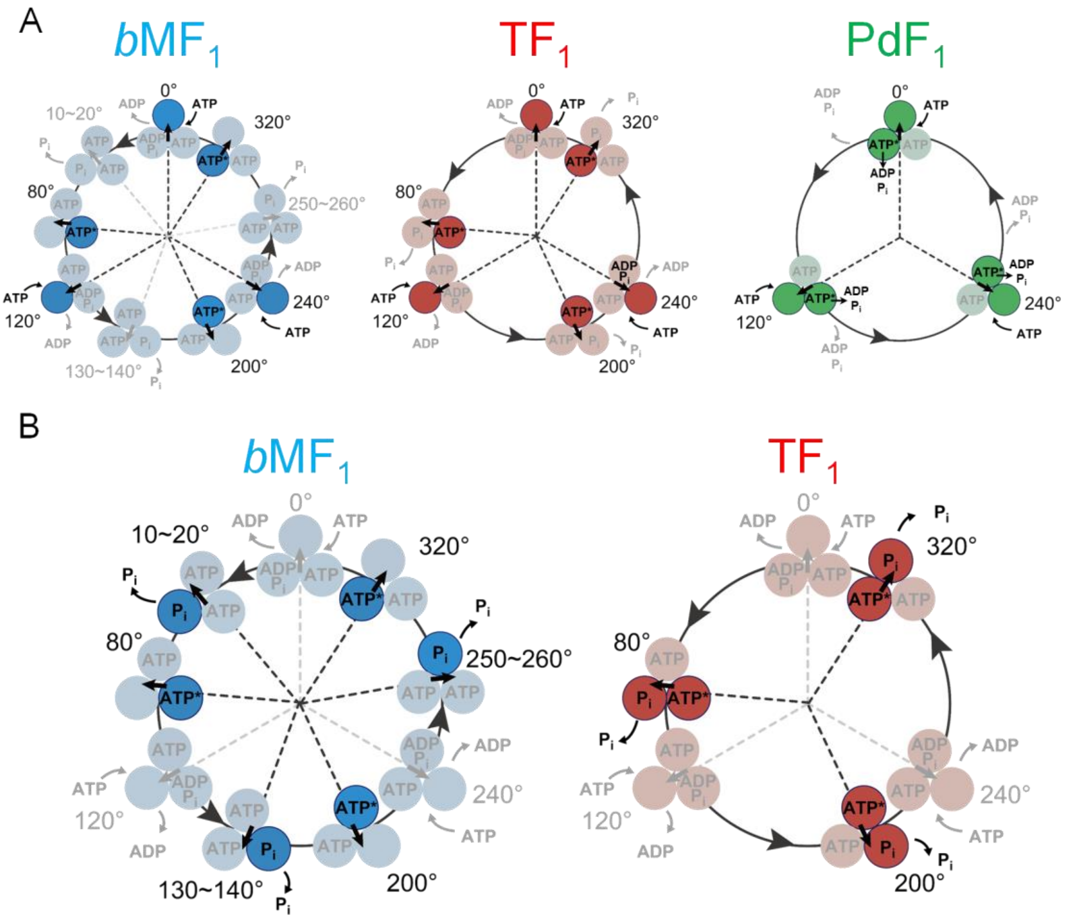


**Figure S7. – Schematic images representing angular potions of rotary substates observed in the rotation assay, related to Figures 3 and 4.**

(*A*) Rotation steps of 3 genuine $F_1$s under low substrate concentration. *Short dwells* are too short to be observed at a time resolution of 0.5 ~ 2 ms. Rotary substates waiting for highlighted reactions, ATP-binding and ATP-cleavage reactions, should be observable as pauses in the rotation assay.

(*B*) Rotation steps of *b*$MF_1$ and $TF_1$ under substrate saturated conditions. The pauses derived from substrate-binding are too short to be observed due to its extremely short time constant at a temporal resolution of 0.1 ms. Rotary substates waiting for highlighted reactions, *catalytic dwells* and *short dwells* reactions, should be observable as pauses in the rotation assay.

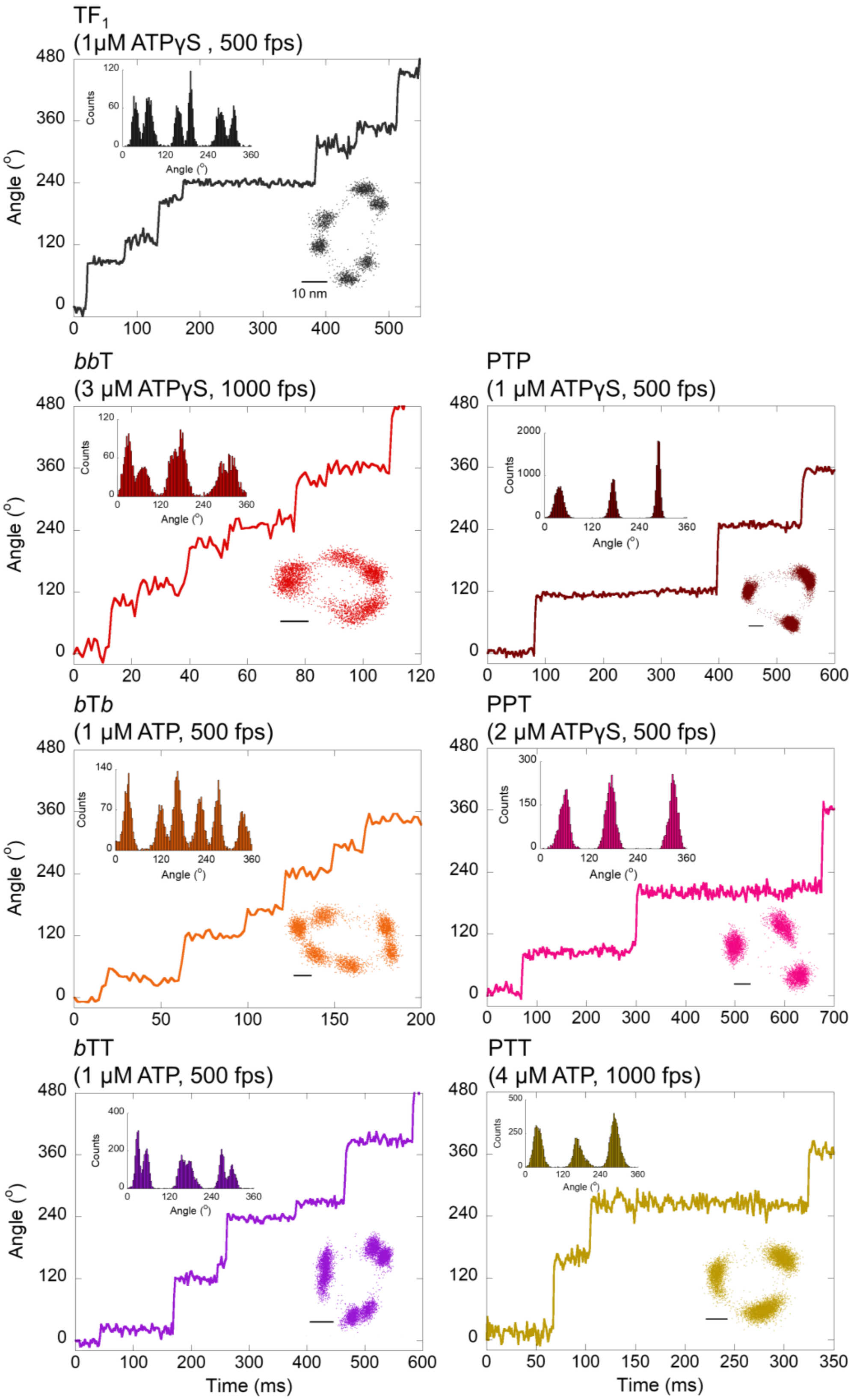


40 **Figure S8. – Rotation trajectories of each $F_1$ under low substrate concentration. (*Upper inset*) histograms of angular positions (Bin width = 3°). (*Lower inset*) *x-y* plot, related to Figure 3.**

42 Experimental condition and frame rate are described in the figure.

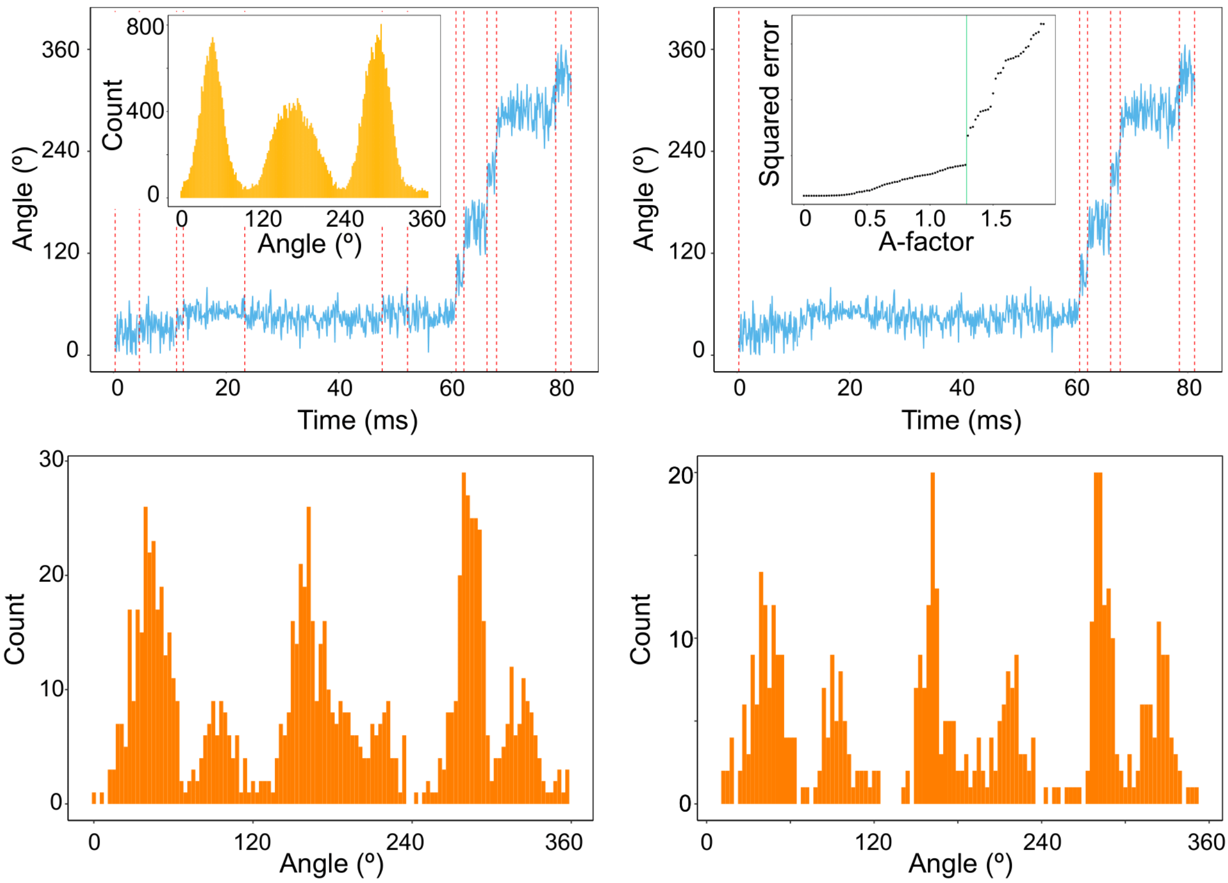


**Figure S9. – Results of change-point analysis before (left column) and after (right column) clean-up procedure, related to Figure 4 and STAR Methods.**

First row of plots shows the angular trace plot of *b*$MF_1$ with vertical change point lines. The inset represents the angular histogram of the angular trace. Inside the plot on the right side, the inset represents the *A* factor vs Squared error plot. In this dataset, 1.2 was chosen as the value of *A* factor. Second row shows the histograms of change point intervals. Consistent with the previous study (Kobayashi *et al.*, 2020), CP analysis successfully detected the short dwells of *b*$MF_1$ around the middle in between two successive *catalytic dwells*.

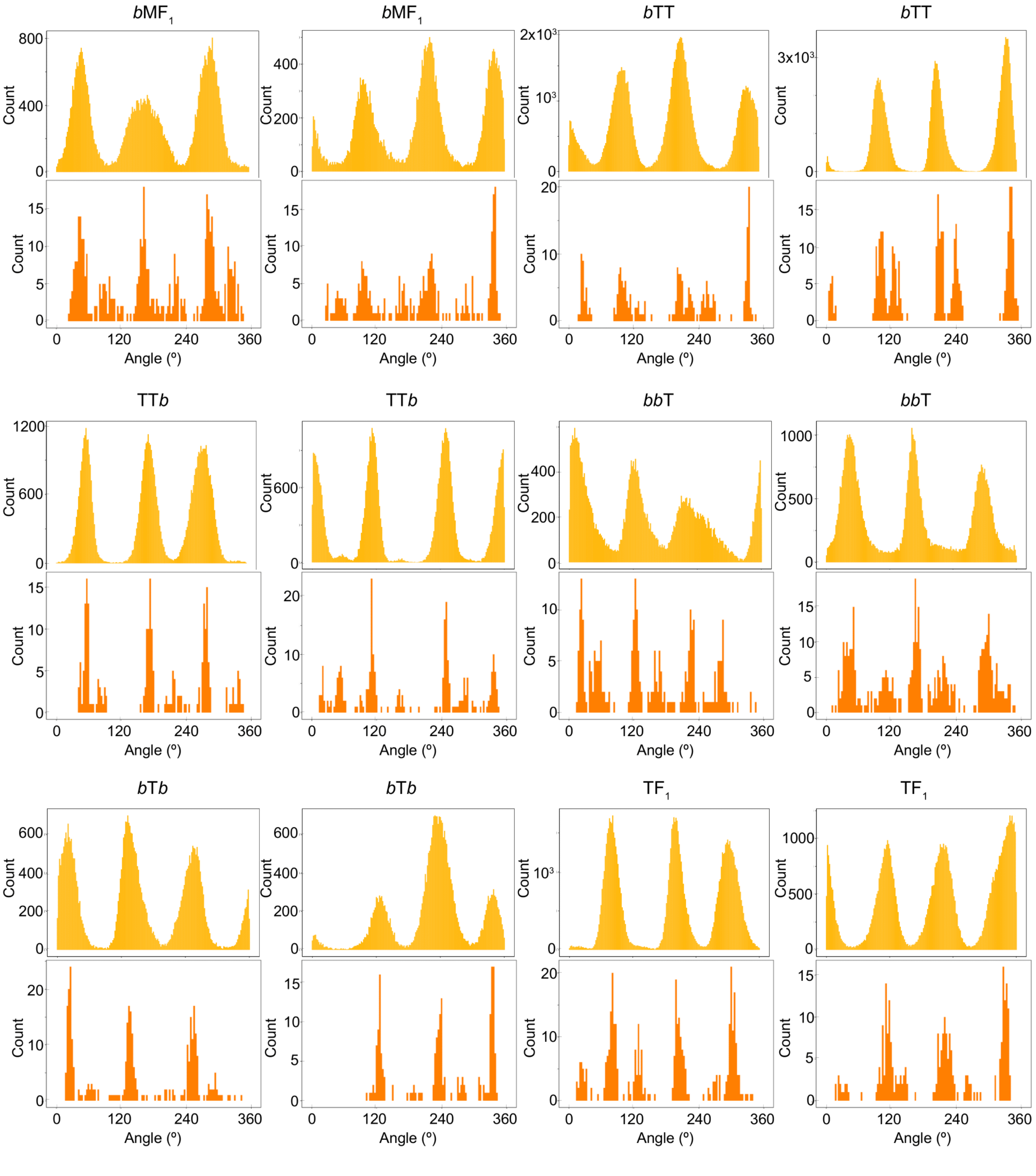


**Figure S10. – Angular histograms (upper) and histograms of change point intervals (lower) for 6-(or 9-) steppers, related to Figure 4.**

After the clean-up procedure, the histograms of the CP intervals were used to detect the number of peaks. Two molecules from each hybrid are selected as examples.

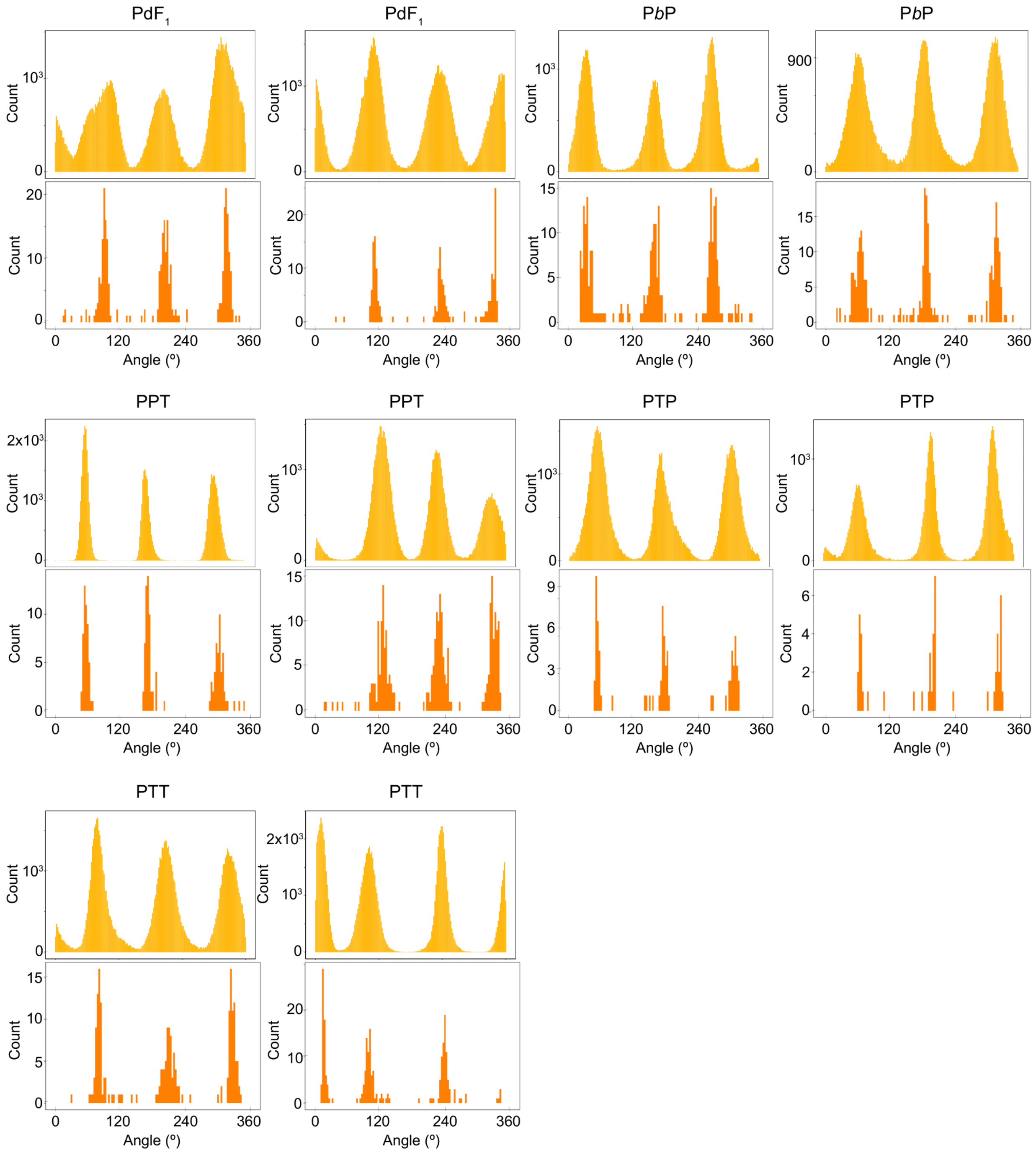


**Figure S11. – Angular histograms (upper) and histograms of change point intervals (lower) for 3-steppers, related to Figure 4.**

After the clean-up procedure, the histograms of the CP intervals were used to detect the number of peaks. Two molecules from each hybrid are selected as examples.

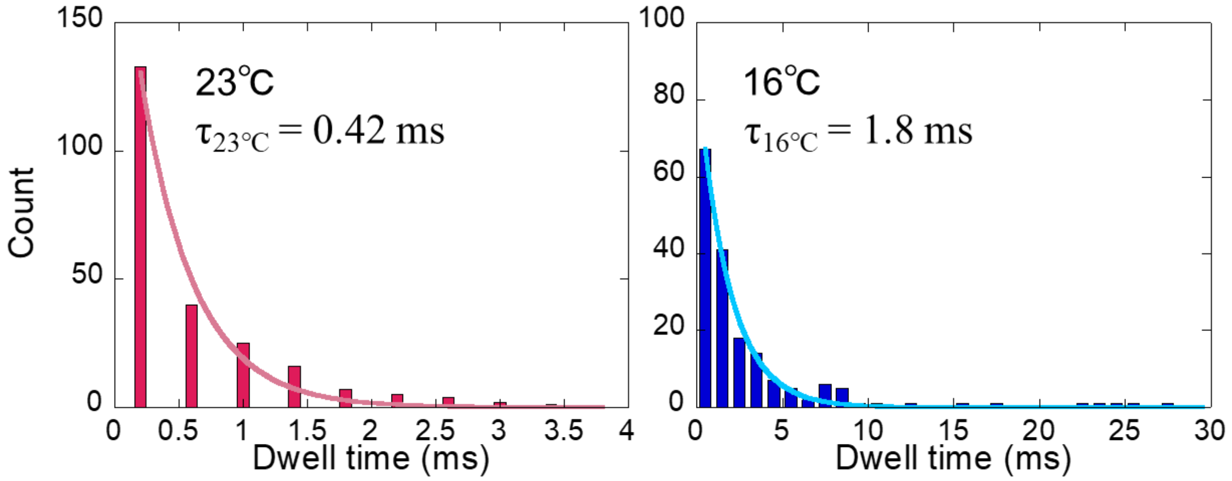


**Figure S12. – Histograms of the dwell times of the short pause in rotation of $TF_1$ in the presence of 1 mM ATPγS at 23 ºC (left) and 16 ºC (right), related to Figure 4.**

Curves were plotted using a first-order reaction scheme, $y = C \cdot exp(-t/\tau)$, where $\tau$ (23 ºC) = 0.42 ± 0.04 ms, and $\tau$ (16 ºC) = 1.8 ± 0.1 ms. Bin width = 0.4 ms (23 ºC) and 1 ms (16 ºC). For 5 molecules, more than 30 dwells were manually extracted from the rotation trajectories, respectively: 4 dwells of the dataset recorded at 16 ºC are outside of the maximum. From these time constants, the $Q_{10}$ factor was estimated to be 8.0.

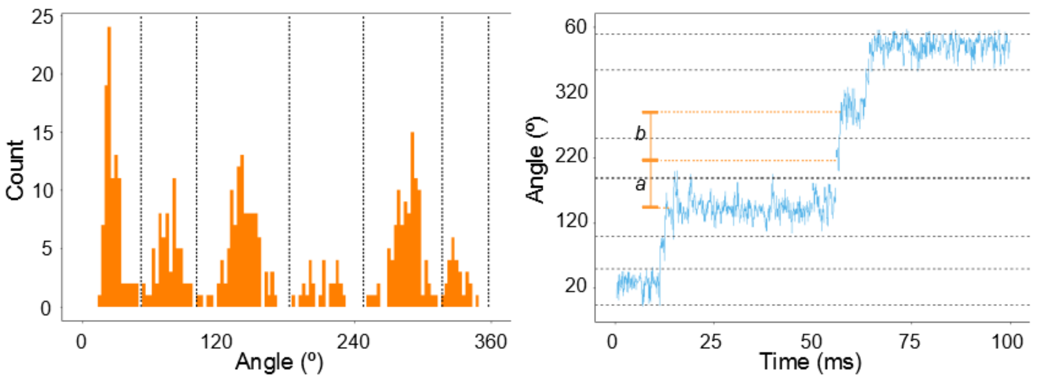


**Figure S13. – Determination of step sizes according to angular trace plot representing angular positions of dwells detected by change-point analysis, related to STAR Methods.**

(Left) A histogram of change point intervals of *b*$MF_1$, as example, showing clear 6 pauses. Dashed lines denote the borders between the 6 pauses assigned manually.

(Right) A section of the angular trace plot of the same molecule with black dashed lines showing the borders selected according to the histogram on the left. Orange dashed lines denote the mean of the change point interval. "*a*" represents the distance between mean angles of the main pause and the following sub-pause, while "*b*" represents the distance between the mean angles of the same sub-pause and the following main pause.

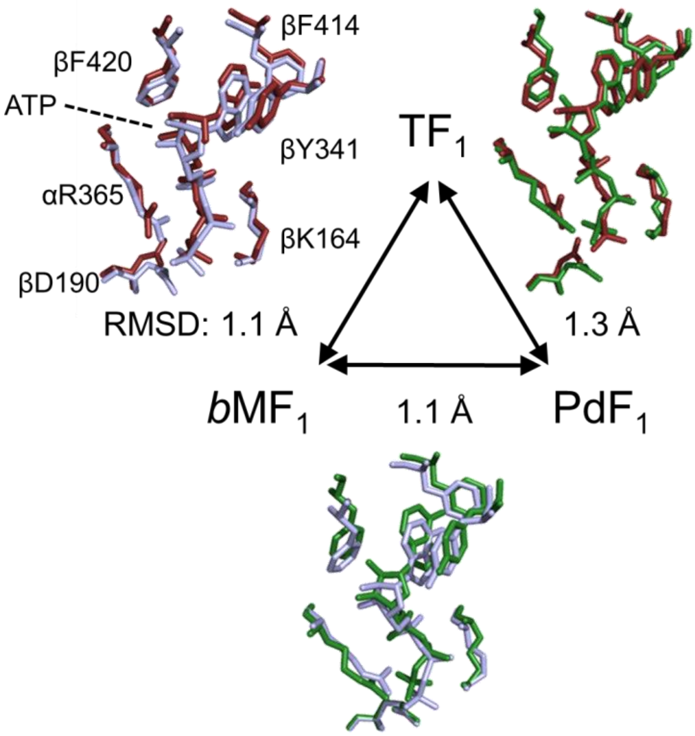


**Figure S14. – Structural comparison of catalytic site among species, related to Figure 2 and Table 2.**

Catalytically important residues around ATP on $β_{DP}$ (αR365, βK164, βD190, βY341, βF414, βF420 in $TF_1$) and the ATP molecules were aligned among $TF_1$ (dark red), *b*$MF_1$ (light blue), and $PdF_1$ (dark green). Black arrows forming a triangle represent the structural comparisons between $TF_1$ and *b*$MF_1$, $TF_1$ and $PdF_1$, or *b*$MF_1$ and $PdF_1$. All-atom RMSD (root-mean-square deviation) values were calculated by alignment of atoms within 1.5 nm from β-phosphate of ATP bound to $β_{DP}$ among the genuine $F_1$s. The small RMSD values suggest that the atomic coordinates of the catalytic residues in the vicinity of ATP are almost identical among genuine $F_1$s. PDB ID: 7L1R for $TF_1$, 2JDI for *b*$MF_1$, and 5DN6 for $PdF_1$ were used.

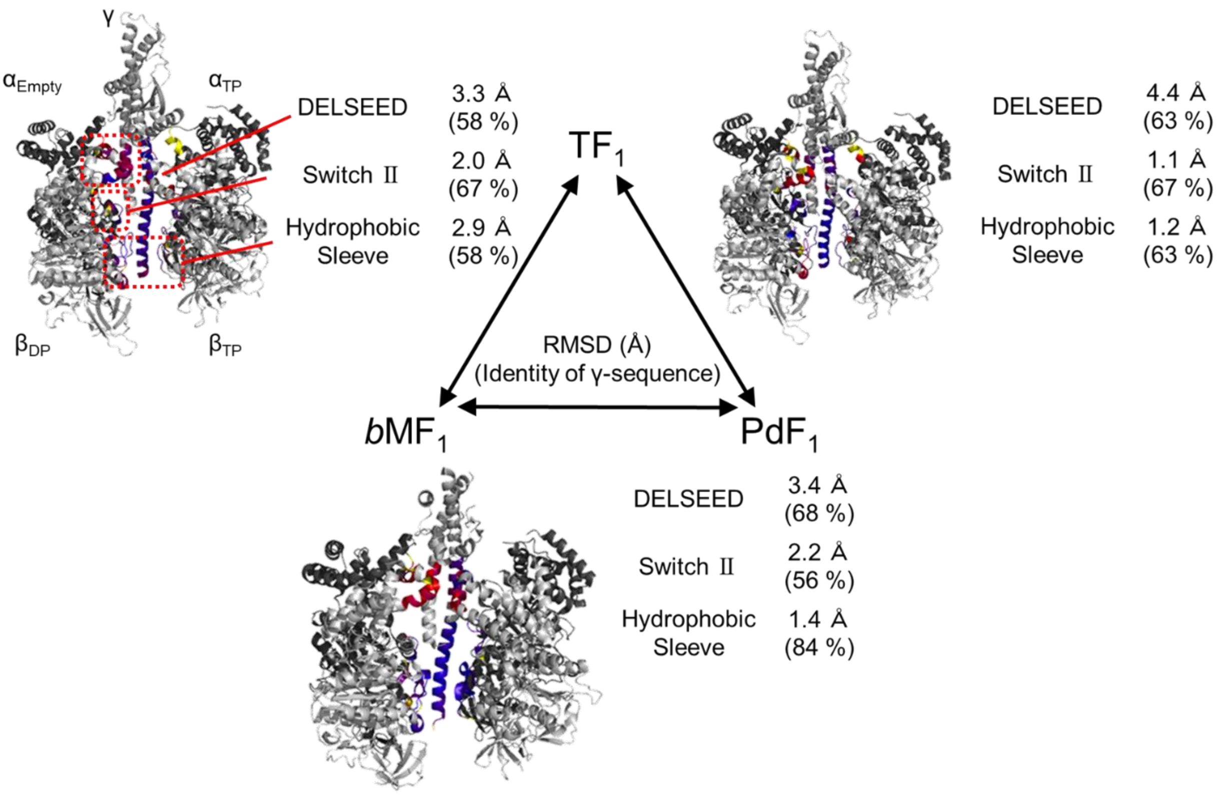


**Figure S15. – Structural comparison of interface between $\alpha_3\beta_3$-ring and γ among species, related to Figures 3 and 4.**

To evaluate the structural similarity of $\alpha_3\beta_3$-γ interface among $TF_1$, *b*$MF_1$, and $PdF_1$, first, we defined the residues of $TF_1$-γ, 18-25 and 243-254 as the portion interacting with DELSEED, 259-267 as the portion interacting with switch II, 267-285 as the portion interacting with hydrophobic sleeve. Also, in *b*$MF_1$-γ and $PdF_1$-γ, corresponding residues were defined as the portions interacting with $\alpha_3\beta_3$ respectively. Then, atoms within 1 nm from each portion of γ are aligned among 3 genuine $F_1$s and the all-atom RMSD values were calculated for each portion. Black arrows forming a triangle represent the structural comparisons between $TF_1$ and *b*$MF_1$, $TF_1$ and $PdF_1$, or *b*$MF_1$ and $PdF_1$. In addition to the all-atom RMSD values, identities of amino acid sequences of γ among species are also described.

In the figure, α subunit is coloured with dark-gray, while β and γ subunits are coloured with light-gray. Then, “blue-red” spectrum (minimum (blue): 0 Å, middle (purple): 1 Å, maximum: 2 Å (red); PyMOL script ColorByRMSD) represents the distance between corresponding atoms. The residues shown in yellow were not used for alignment. For clarity, only structure of $TF_1$ or $PdF_1$ was shown in the figure. Also, $\alpha_{DP}$ in the front side and $\beta_{Empty}$ in the back side are not shown. DELSEED region shows relatively high RMSD values in the structural alignment among species compared with hydrophobic sleeve region or switch II region, even though conservation of amino acid sequence for each region is similar among species. PDB ID: 7L1R for $TF_1$, 2JDI for *b*$MF_1$, and 5DN6 for $PdF_1$ were used.

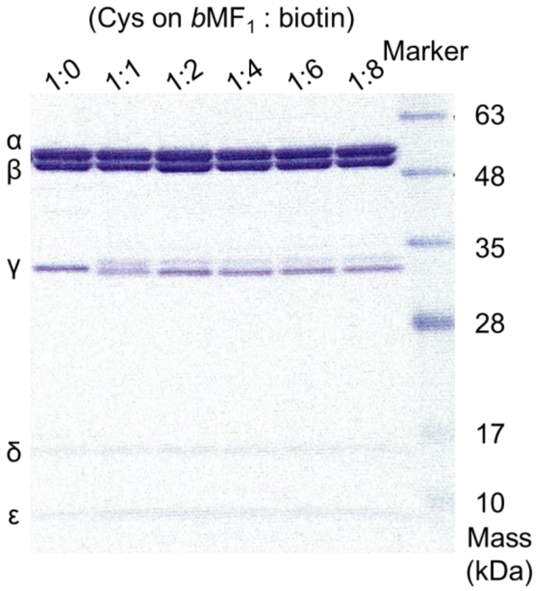


**Figure S16. – SDS-PAGE analysis of biotinylated *b*MF$_1$, related to STAR Methods.**

Each lane corresponds to the sample consisting of 0.5 μM *b*MF$_1$ and biotin (0 ~ 8 μM).

**Table S1. Rotation scheme of $TF_1$, related to Figure 1.**

Angular positions of the γ subunit of $TF_1$ are summarized in the leftmost column. Elementary reactions of ATP hydrolysis occurring on each β subunit are described in the other columns. The angular position of γ where ATP-binding occurs on $\beta_1$ is defined as 0º.

| γ | $\beta_1$ | $\beta_2$ | $\beta_3$ |
|---|---|---|---|
| 0º | ATP-binding | ADP-release | |
| 80º | | $P_i$-release | Cleavage |
| 120º | | ATP-binding | ADP-release |
| 200º | Cleavage | | $P_i$-release |
| 240º | ADP-release | | ATP-binding |
| 320º | $P_i$-release | Cleavage | |

**Table S2. Classification of rotation schemes among species, related to Figures 1, 3, and 4.**

The number of rotational steps per turn was analyzed at various concentrations of ATP or ATPγS.

| | *b*$MF_1$-type | $TF_1$-type | $PdF_1$-type |
|---|---|---|---|
| Sub-steps / 120º rotation | 3<br>BP, CP, SP | 2<br>BP, CP | 1<br>BP & CP |
| Number of pauses<br>at [ATP]s near $K_m$ | 2<br>BP, CP | 2<br>BP, CP | 1<br>BP & CP |
| Number of pauses<br>at high [ATP]s | 2<br>CP, SP | 1 (2*)<br>CP, BP | 1<br>BP & CP |
| Angular distance ratio | 1<br>(= 60º / 60º) | 0.5<br>(= 40º / 80º) | -<br>- |

BP: *Binding dwell*

CP: *Catalytic dwell*

SP: *Short dwell*

*: In present study, 2 dwells were observed, contrary to expectations

**Table S3. Compositions of hybrid $F_1$, related to Figure 1.**

| Hybrid | Origin of Subunit α | β | γ | $\delta^{bacteria}$ | $\varepsilon^{bacteria}$ | $\delta^{b}$ | $\varepsilon^{b}$ | Vector | Purification Rotation |
|---|---|---|---|---|---|---|---|---|---|
| *bb*T | *b* | *b* | T | - | - | *b* | *b* | *b* | *** |
| TT*b* | T | T | *b* | - | - | *b* | *b* | *b* | *** |
| *b*T*b* | *b* | T | *b* | - | - | *b* | *b* | *b* | *** |
| *b*TT | *b* | T | T | - | - | *b* | *b* | *b* | *** |
| T*b*T | T | *b* | T | -<br>- | -<br>- | -<br>*b* | -<br>*b* | T<br>*b* | *<br>** |
| T*bb* | T | *b* | *b* | - | - | *b* | *b* | *b* | * |
| P*b*P | P | *b* | P | P | P | - | - | P | *** |
| PPT | P | P | T | P | P | - | - | P | *** |
| PTP | P | T | P | P | P | - | - | P | *** |
| PTT | P | T | T | P | P† | - | - | P | *** |
| PP*b* | P | P | *b* | -<br>P | -<br>P | *b*<br>- | *b*<br>- | *b*<br>P | *<br>* |
| *b*P*b* | *b* | P | *b* | - | - | *b* | *b* | *b* | ** |

***: Rotary catalysis was confirmed.

**: Purification was successful though rotation assay was not successful.

*: Purification was not successful.

†: In the SDS-PAGE gel (Figure S3), a band of shorter fragment than ε from $PdF_1$ was observed.

"*b*", "T", and "P" represent $b$$MF_1$, $TF_1$ and $PdF_1$ respectively.

In the lanes of δ and ε, letters are written in red if the corresponding bands were observed in the SDS-PAGE analysis (Figure S3).

**Table S4. Michaelis-Menten parameters in ATPγS-driven rotation of the genuine and hybrid $F_1$s (*n* = 5), related to Figure 4 and Table 1.**

Michaelis-Menten parameters, $V_{max}^{ATP\gamma S}$ and $K_m^{ATP\gamma S}$ of the $F_1$s estimated from Fig S5C. They are shown as fitted parameter ± fitting error.

| | $V_{max}^{ATP\gamma S}$ (rps) | $K_m^{ATP\gamma S}$ (μM) |
|---|---|---|
| *b*MF$_1$ | 22 ± 0.5 | 4.5 ± 0.6 |
| TF$_1$ | 9.4 ± 0.3 | 0.6 ± 0.1 |
| PdF$_1$ | 5.9 ± 0.2† | 1.8 ± 0.3† |
| *bb*T | 15 ± 0.5 | 3 ± 0.5 |
| P*b*P | 13 ± 0.5 | 15 ± 3 |
| PPT | 1.6 ± 0.1 | 1.6 ± 0.4 |
| PTP | 2.6 ± 0.1 | 2.3 ± 0.5 |
| TT*b* | - | - |
| *b*T*b* | - | - |
| PTT | - | - |
| *b*TT | - | - |

“-”: Not determined

†: Zarco-Zavala *et al.,* 2020

**Table S5. The mean values of angular distance ratios *a / b* of $TF_1$, *b*$MF_1$, and $TF_1$-*b*$MF_1$ hybrids, related to Figure 4.**

Angular distances were calculated from the mean angular distance ratio.

| | Number of CP intervals | Mean of *a / b* | Angular distance |
|---|---|---|---|
| *b*TT | 380 | 0.36 | 32º:88º |
| $TF_1$ | 454 | 0.63 | 46º:74º |
| *bb*T | 880 | 0.70 | 49º:71º |
| TT*b* | 334 | 0.87 | 56º:64º |
| *b*$MF_1$ | 644 | 0.95 | 58º:62º |
| *b*T*b* | 539 | 1.21 | 66º:54º |

**Table S6. Kinetic parameters of genuine and hybrid $F_1$s, related to Table 1.**

| $F_1$ | Origin of subunits | | | Vector | Purification Rotation | $V_{max}^{ATP}$ (rps) | $K_m^{ATP}$ (μM) | $V_{max}^{ATP\gamma S}$ (rps) | $K_m^{ATP\gamma S}$ (μM) | Pause |
|---|---|---|---|---|---|---|---|---|---|---|
| | α | β | γ | | | | | | | |
| *b*$MF_1$ | *b* | *b* | *b* | *** | *** | 786 ± 4 | 67 ± 1 | 22 ± 0.5 | 4.5 ± 0.6 | 9 |
| $TF_1$ | T | T | T | *** | *** | 189 ± 2 | 17 ± 1 | 9.4 ± 0.3 | 0.6 ± 0.1 | 6 |
| $PdF_1$ | P | P | P | *** | *** | 338 ± 5 | 77 ± 4 | 5.9 ± 0.2 | 1.8 ± 0.3 | 3 |
| *bb*T | *b* | *b* | T | *b* | *** | 608 ± 3 | 61 ± 2 | 15 ± 0.5 | 3 ± 0.5 | *b*/T |
| TT*b* | T | T | *b* | *b* | *** | 57 ± 3 | 8 ± 2 | - | - | *b*/T |
| *b*T*b* | *b* | T | *b* | *b* | *** | 17 ± 1 | 2 ± 0 | - | - | *b*/T |
| *b*TT | *b* | T | T | *b* | *** | 5 ± 0 | 1 ± 0 | - | - | *b*/T |
| T*b*T | T | *b* | T | T<br>*b* | *<br>** | | | | | |
| T*bb* | T | *b* | *b* | *b* | * | | | | | |
| P*b*P | P | *b* | P | P | *** | 477 ± 21 | 117 ± 19 | 13 ± 0.5 | 15 ± 3 | 3 |
| PP*b* | P | P | *b* | *b*<br>P | *<br>* | | | | | |
| *b*P*b* | *b* | P | *b* | *b* | ** | | | | | |
| P*bb* | P | *b* | *b* | | - | | | | | |
| *bb*P | *b* | *b* | P | | - | | | | | |
| *b*PP | *b* | P | P | | - | | | | | |
| PPT | P | P | T | P | *** | 228 ± 4 | 59 ± 5 | 1.6 ± 0.1 | 1.6 ± 0.4 | 3 |

| | | | | | | | | | | |
|---|---|---|---|---|---|---|---|---|---|---|
| PTP | P | T | P | P | *** | 123 ± 1 | 33 ± 2 | 2.6 ± 0.1 | 2.3 ± 0.5 | 3 |
| PTT | P | T | T | P | *** | 9 ± 1 | 4 ± 2 | - | - | 3 |
| TPP | T | P | P | | - | | | | | |
| TPT | T | P | T | | - | | | | | |
| TTP | T | T | P | | - | | | | | |

*** : Rotary catalysis was confirmed.

** : Purification was successful though rotation assay was not successful.

* : Purification was not successful.

- : Not attempted.

"*b*", "T", and "P" represent *b*$MF_1$, $TF_1$, and $PdF_1$ respectively. First 3 rows represent 3 genuine $F_1$s. 4th ~ 9th rows represent hybrid $F_1$s between *b*$MF_1$ and $TF_1$. 10th ~ 15th rows represent hybrid $F_1$s between *b*$MF_1$ and $PdF_1$. 16th ~ 21th rows represent hybrid $F_1$s between $PdF_1$ and $TF_1$. The errors represent fitting errors.

**Table S7. Sequences of the forward (F) and reverse (R) primers for $TF_1$-*b*$MF_1$ hybrid $F_1$s, related to STAR Methods.**

| | | | |
|---|---|---|---|
| $TF_1$ | α | F | TCTAGAGTCGACTAAGAAGGAGATCATCATATGAGCCATCATCATCATCATCATGG |
| | | R | TTCTCAACCGGTTTATTGTGAAACGACAAACGTTTTCTTGAAC |
| | β | F | GTAAGCGCGGCCGCTAAGAAGGAGATATCCATATGCATCATCATCATCATCATCATCATCATCATATGACAAG |
| | | R | TTCTCACGCCGGCGTCACACTTCGACACCCATCGCTTTCGC |
| | γ | F | GCCTAAACCGGTTGAGAAGGAGATATACATATGGCATCGTTACGCGATATTAAAACG |
| | | R | TTCTTAGCGGCCGCGCTTATTGCAAGGCGTTTGCTCCG |

**Table S8. Sequences of the forward (F) and reverse (R) primers for $PdF_1$-hybirds, related to STAR Methods.**

| PTP | Sequence |
|---|---|
| $F_1\ \beta^{Hyb}$ | CAAGAGCTGCAGGCACAGGGCA |
| $R_1\ \beta^{Hyb\text{-}T}$ | GCGTCCTCTTGTCATGTCTCTACCTCTACGTGTCAGAGCGCC |
| $F_2\ \beta^{Hyb\text{-}T}$ | GTAGAGGTAGAGACATGACAAGAGGACGCGTTATCCAAG |
| $R_2\ \beta^{Hyb\text{-}T}$ | CGCCCCCTTCACACTTCGACACCCATCGC |
| $F_3\ \beta^{Hyb\text{-}T}$ | TCGAAGTGTGAAGGGGGCGACATGGCCGAC |
| $R_3\ \beta^{Hyb}$ | TCATTCTAGACTAATGCGGGAAATTCGCAT |

| PPT | Sequence |
|---|---|
| $F_1\ \gamma^T$ | TACGCCTGAGGGGAGGGATAGATGGCATCGTTACGCGATATT |
| $R_1\ \gamma^T$ | GATGATGCATGTCTCTACCTCTACGTGTTATTGCAAGGCGTTTGCTC |
| $F_2\ \gamma^T$ | CACGTAGAGGTAGAGACATGCATCATCACC |
| $R_2\ \gamma^T$ | GAGACGCTGAGCCTTGGCTTTCAC |

| PTT | Sequence |
|---|---|
| $F_1\ \beta\text{-}\gamma^T$ | TACGCCTGAGGGGAGATGGCATCGTTACGCGATATTAAAAC |
| $R_2\ \beta^{Hyb\text{-}T}$ | CGCCCCCTTCACACTTCGACACCCATCGC |

| P*b*P | Sequence |
|---|---|
| $F_1\ \beta^{Hyb}$ | CAAGAGCTGCAGGCACAGGGCA |
| $R_1\ \beta^{Hyb\text{-}bM}$ | ATGATGATGATGCATGTCTCTACCTCTACGTGTCAGAGCGCCTCT |
| $F_2\ \beta^{Hyb\text{-}bM}$ | ACGTAGAGGTAGAGACATGCATCATCATCATCATCATCA |
| $R_2\ \beta^{Hyb\text{-}bM}$ | CGCCCCCTTCATGAGTGCTCTTCAGCCAA |
| $F_3\ \beta^{Hyb\text{-}bM}$ | AGCACTCATGAAGGGGGCGACATGGCCGAC |
| $R_3\ \beta^{Hyb}$ | TCATTCTAGACTAATGCGGGAAATTCGCAT |
